# QSAR and molecular docking studies of isatin and indole derivatives as SARS 3CL^pro^ inhibitors

**DOI:** 10.1186/s13065-023-00947-w

**Published:** 2023-04-07

**Authors:** Niousha Soleymani, Shahin Ahmadi, Fereshteh Shiri, Ali Almasirad

**Affiliations:** 1 Department of Medicinal Chemistry, Faculty of Pharmacy, Tehran Medical Sciences, Islamic Azad University, Tehran, Iran; 2 Department of Chemistry, Faculty of Pharmaceutical Chemistry, Tehran Medical Sciences, Islamic Azad University, Tehran, Iran; 3 Department of Chemistry, University of Zabol, Zabol, Iran

**Keywords:** QSAR, Molecular docking, Isatin derivatives, Indole derivatives, SARS CoV 3CL^pro^ inhibitor, Index of ideality of correlation

## Abstract

The 3C-like protease (3CL^pro^), known as the main protease of SARS-COV, plays a vital role in the viral replication cycle and is a critical target for the development of SARS inhibitor. Comparative sequence analysis has shown that the 3CL^pro^ of two coronaviruses, SARS-CoV-2 and SARS-CoV, show high structural similarity, and several common features are shared among the substrates of 3CL^pro^ in different coronaviruses. The goal of this study is the development of validated QSAR models by CORAL software and Monte Carlo optimization to predict the inhibitory activity of 81 isatin and indole-based compounds against SARS CoV 3CL^pro^. The models were built using a newer objective function optimization of this software, known as the index of ideality correlation (IIC), which provides favorable results. The entire set of molecules was randomly divided into four sets including: active training, passive training, calibration and validation sets. The optimal descriptors were selected from the hybrid model by combining SMILES and hydrogen suppressed graph (HSG) based on the objective function. According to the model interpretation results, eight synthesized compounds were extracted and introduced from the ChEMBL database as good SARS CoV 3CL^pro^ inhibitor. Also, the activity of the introduced molecules further was supported by docking studies using 3CL^pro^ of both SARS-COV-1 and SARS-COV-2. Based on the results of ADMET and OPE study, compounds CHEMBL4458417 and CHEMBL4565907 both containing an indole scaffold with the positive values of drug-likeness and the highest drug-score can be introduced as selected leads.

**Supplementary Information:**

The online version contains supplementary material available at 10.1186/s13065-023-00947-w.

## Introduction

In the end of February 2003, a novel human coronavirus was detected as the causative agent of the first major pandemic of the twenty-first century, severe acute respiratory syndrome (SARS). The first case of "atypical pneumonia" was declared in China and quickly and unexpectedly spread to 29 countries, especially in Asia and North America, alarming the World Health Organization (WHO). Within several months of the outbreak in 2003, the WHO reported that it had caused 916 deaths out of 8422 cases worldwide (10–15% case fatality rate) [1]. In early 2003, a new human coronavirus known as SARS coronavirus (SARS CoV) was recognized as the causative agent of SARS [2].

COVID-19 is the active pandemic which was first reported in late 2019 in Wuhan, China. In February 2020, SARS-COV-2 was announced as the causative agent. As of October 24th 2021, 243 million cases and over 4.9 million deaths have been reported. The 3C-like protease (3CL^pro^) enzyme or major protease (M^pro^), is essential for the process of viral replication and infection, thereby making it an ideal target for antiviral therapy [1]. The coronavirus 3CL^pro^ is a cysteine protease consisting of about 300 amino acids and containing three domains. Domains I (amino acids 8 to 99) and II (amino acids 100 to 183) consist of beta barrels that simulate the chymotrypsin and 3C proteinases. The binding site is located between the mentioned domains, and about 16 residues join domains I and II to residues 200 to 300 as the C-terminal domain III. The proteolytic activity of 3CL^pro^ has been performed by this third five helices domain [3]. The 3CLpro enzymes show a highly conserved structure among known coronavirus species, and several common characteristics are shared among different coronavirus 3CLpro substrates [4]. Comparative sequence analysis has shown that the 3CLpros of the three coronaviruses of SARS-CoV-2, SARS-CoV, and MERS-CoV are very similar in structure and conservatism [5]. These findings indicate that 3CLpro could be used as a homologous target for the development of anti-coronavirus drugs that can inhibit the proliferation of various coronaviruses [4].

Based on various studies, a combination of nucleoside analogues such as ribavirin can be used for the treatment of SARS along with corticosteroids such as methylprednisolone and hydrocortisone [6–9]. Since the beginning of the COVID-19 pandemic different options for the treatment of this disease have been used including monoclonal antibodies, protease inhibitors, corticosteroids, convalescent plasma and so on. However, the definitive efficacy of these drugs has not been proven.

Previous research has revealed that isatin and its derivatives have a broad range of anti-bacterial and anti-viral activities such as anti-HIV [10, 11], anti-rhinovirus [12] and against mycobacterium tuberculosis [13]. The derivatized isatin scaffold may be a good candidate for the SARS CoV 3CL^pro^ inhibitor because both proteases (human SARS CoV and rhinovirus) are cysteine proteases and are structurally similar in the active site [14].

In 2005, Chen et al. investigate that N-substituted isatin derivatives with anti-rhinovirus activity may also have anti-SARS activity. Therefore, based on these compounds, they synthesized new isatin derivatives and evaluated their inhibition activities against SARS CoV 3CL^pro^. The IC_50_ values showed that the mentioned isatin derivatives could inhibit SARS CoV 3CL^pro^ in the low micro molar range (0.95–17.50 µM) [15]. Using the results of the previous study, Zhou et al. designed and synthesized a series of N-substituted 5-carboxamide-isatin compounds and evaluated their activities. They introduced some compounds as SARS CoV 3CL^pro^ inhibitors which the most potent compound showed an IC_50_ of 0.37 µM [2]. In 2014 Liu et al. in order to improve the inhibitory activity of isatin derivatives against SARS CoV 3CL^pro^, investigated a replacement of the carboxamide group using a series of substituted sulfonamide groups in isatin. Optimization of 5-sulfonyl isatin derivatives led to the discovery of a new compound with the strongest potency (IC_50_ = 1.04 µM) [16].

Quantitative structure–activity relationship (QSAR) is one of the critical computational techniques for ligand-based drug design, which can statistically show the correlation between the structural and bioactive properties of compounds [17]. Molecular docking is a computational technique for predicting the optimal interaction of two molecules that creates a binding model, typically a small ligand with a protein receptor [18], most commonly used in drug discovery [19]. CORAL is a new software for developing the reliable and predictive QSAR/QSPR models based on SMILES or quasi-SMILES of materials and Monte Carlo optimization [17, 20].

The main goal of this study is to create the simple and reliable QSAR models by CORAL software to predict the inhibitory activity of 81 isatin and indole-based compounds against SARS CoV 3CL^pro^. In addition, the effect of using the index of ideality correlation (IIC) as the objective function for modeling in CORAL software has been investigated [21]. Moreover, the results from Monte Carlo optimization-based QSAR modeling with the further addition of molecular docking studies applied for pharmacologically important endpoints. SMILES notation-based optimal descriptors, defined as molecular fragments, identified as main contributors to the increase/decrease of biological activity, which are used further to search compounds from the ChEMBL database with targeted activity based on computer calculation, are presented. Here, molecular docking was applied as an additional method to validate the calculated activity of proposed compounds as novel SARS CoV 3CL^pro^ inhibitors.

## Data and methods

### Dataset

In this study 81 isatin and indole-based SARS 3CLpro inhibitors were gathered from literature [2, 15, 16, 22–25]. The number isatin based compounds were 41 and the rest were indole-based compounds. The IC_50_ (µM) values for inhibitors were converted into their pIC_50_ (− logIC_50_). Table 1 shows the structure of the molecules along with their pIC_50_ (range between 4.08 and 7.77). BIOVIA Draw 2020 was used to draw the molecular structures of the compounds and convert them into SMILES symbols. The dataset divided the active training (≈25%), passive training (≈20%), calibration (≈20%), and validation (≈35%) sets randomly. To construct the QSAR models based on Monte Carlo optimization, four separate random partitions were performed.

**Table 1:**
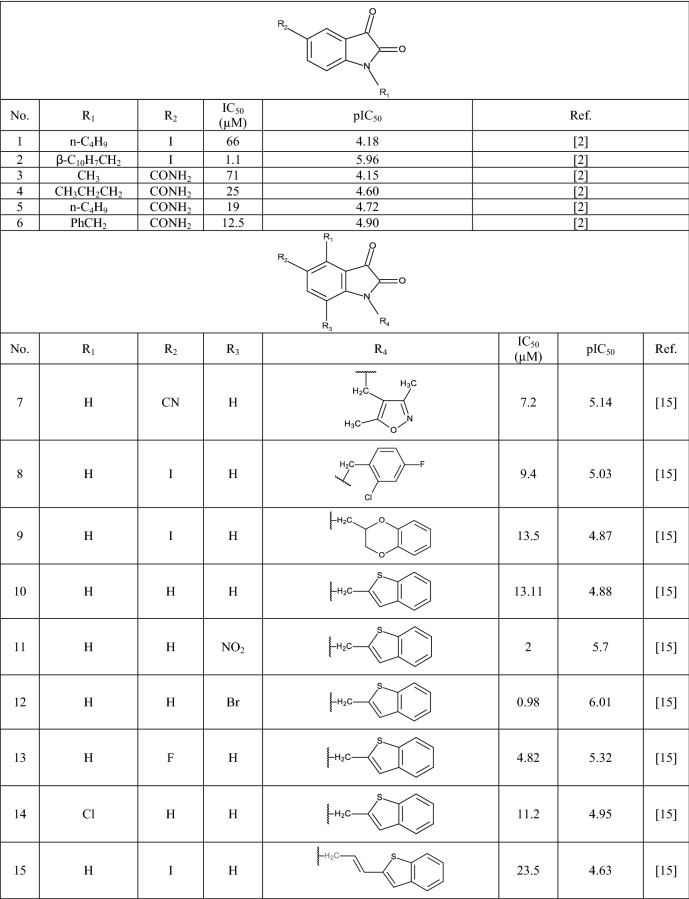

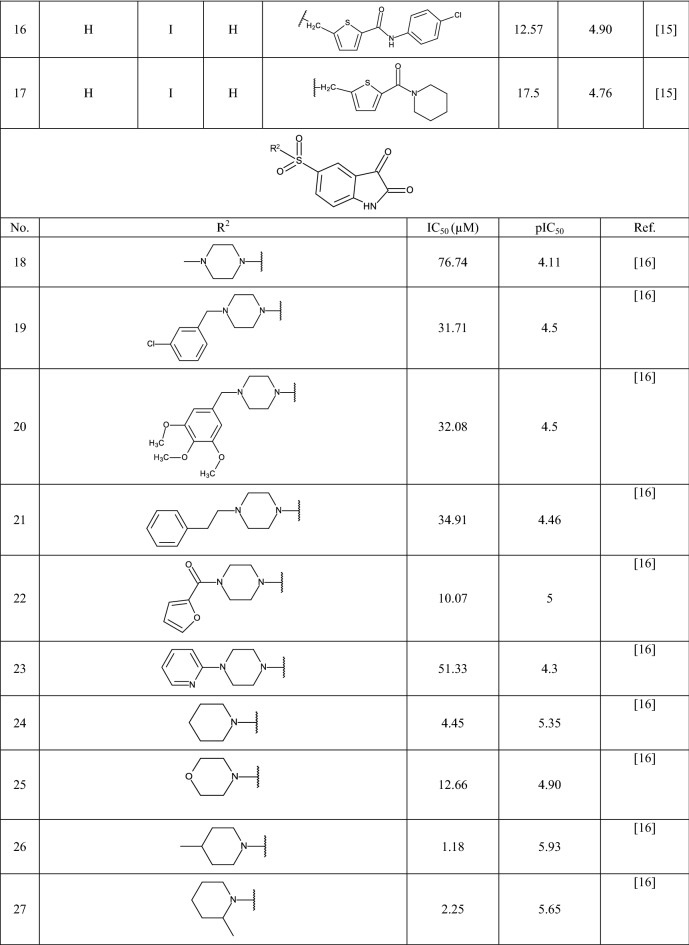

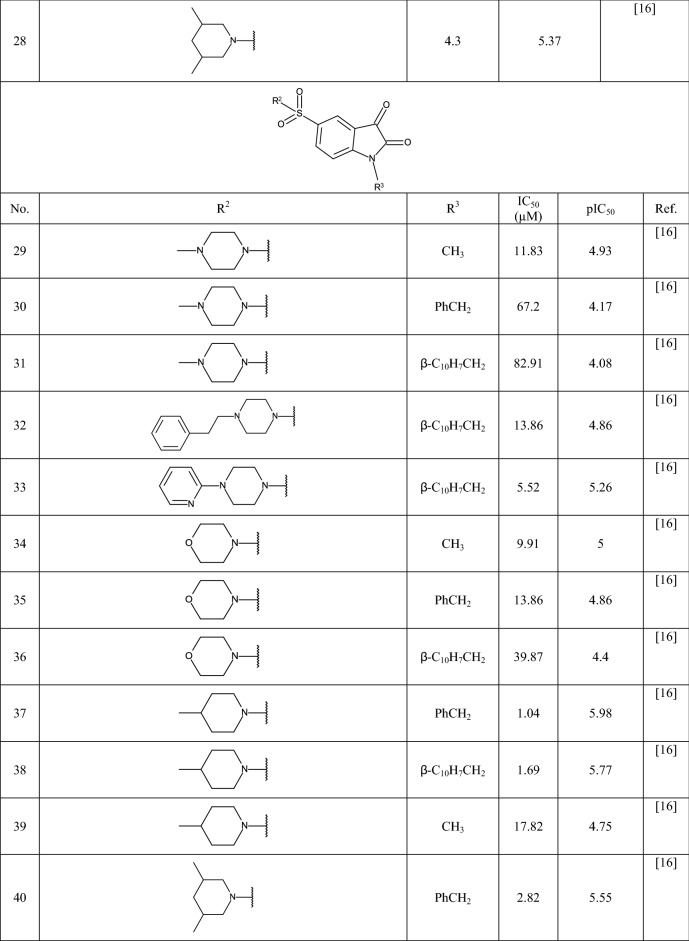

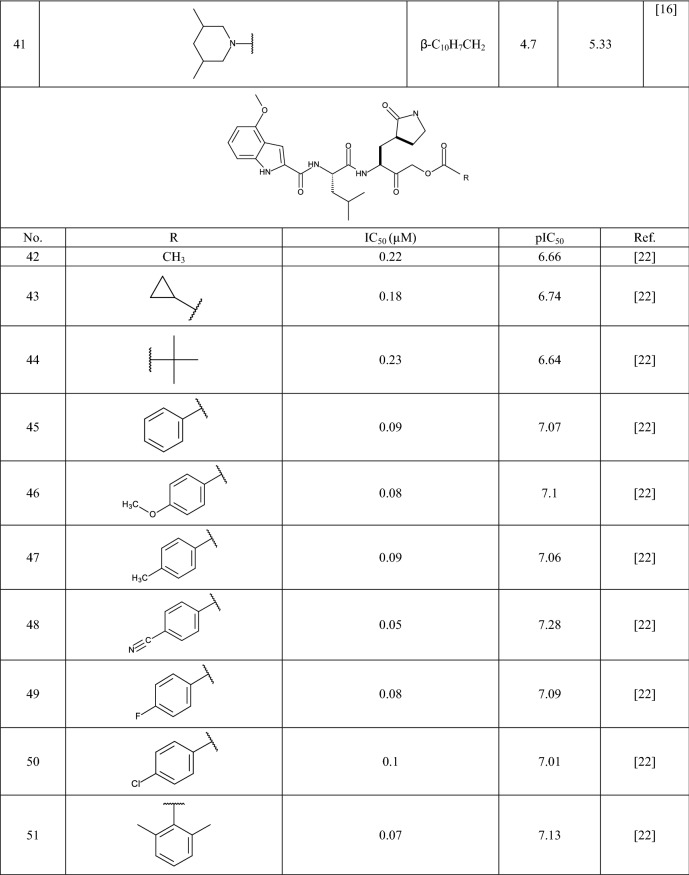

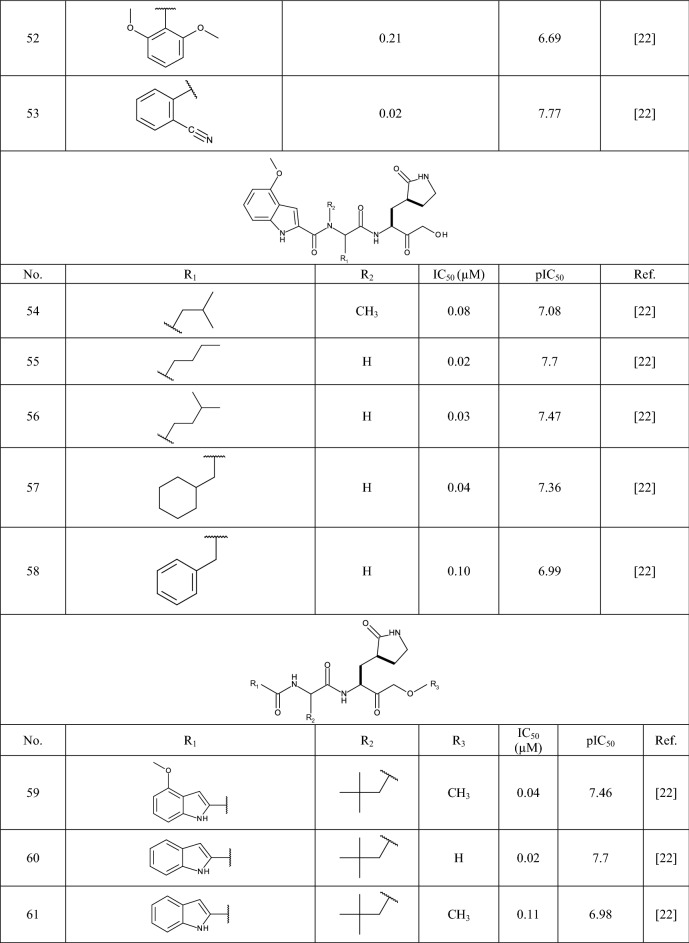

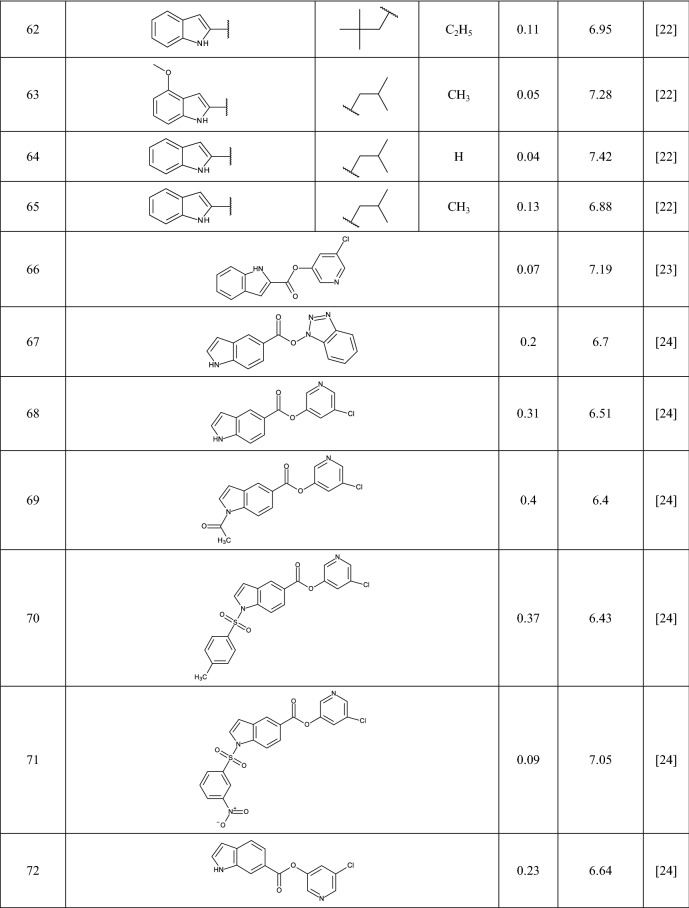

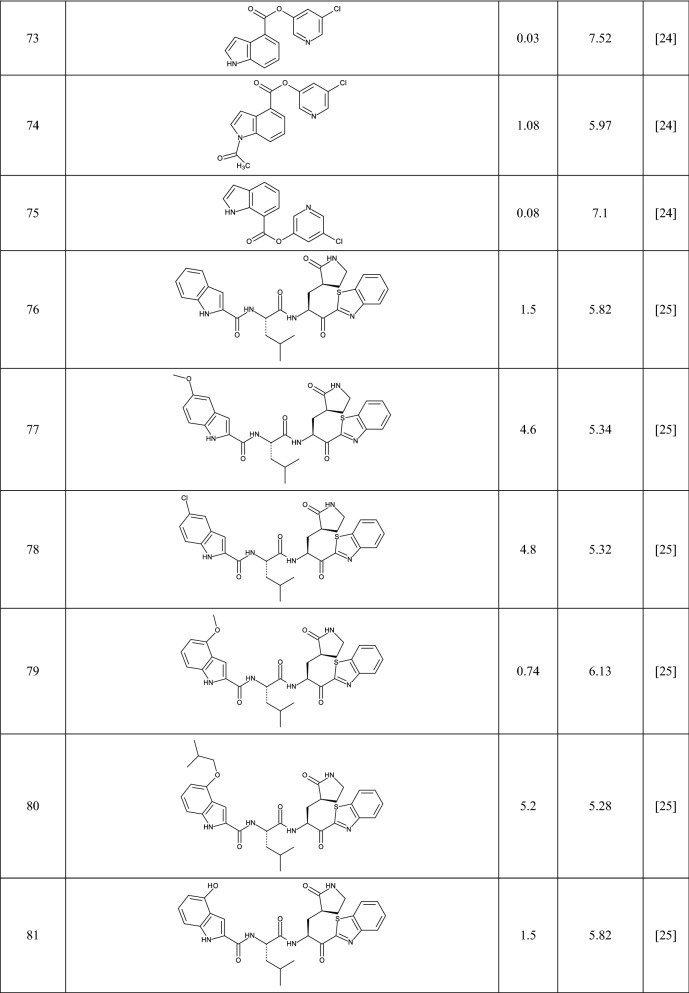
Molecular structures of isatin and indole derivatives along with their pIC_50_

### Descriptors

There are three categories of optimal descriptors in CORAL software, including SMILES-based, graph-based and a combination of SMILES with molecular graph descriptors as hybrid descriptors. The optimal descriptors used in this research to construct the QSAR model are a combination of hydrogen suppression graph (HSG) and SMILES descriptors. The below equation indicates the optimal type of molecular descriptors for QSAR modeling for pIC_50_ of isatin and indole-based compounds as SARS 3CL^pro^ inhibitors:

1 $$\text{DCW}\left(\text{T},\text{ N}\right)=\sum \text{CW}\left({\text{S}}_{\text{k}}\right) +\sum \text{CW}\left({\text{SS}}_{\text{k}}\right)+\sum \text{CW}\left({\text{SSS}}_{\text{k}}\right)+\text{CW}\left(\text{BOND}\right)+\text{CW}\left(\text{NOSP}\right)+\text{CW}\left(\text{HALO}\right)+\text{CW}\left(\text{HARD}\right)+\text{CW}\left(\text{PAIR}\right)+\text{CW}\left(\text{Cmax}\right)+\text{CW}\left(\text{Nmax}\right)+\text{CW}\left(\text{Omax}\right)+\text{CW}\left(\text{Smax}\right)+\text{CW}\left(\text{C}5\right)+\text{CW}\left(\text{C}6\right)$$

where, Sk, SSk and SSk are one, two and three-character SMILES features, respectively. BOND represents a global SMILES descriptor that demonstrate the presence/absence of various bonds including double ( =), triple (#), and stereochemical (@) bonds. The NOSE indicates the presence/absence of nitrogen, oxygen, sulfur, and phosphorus atoms in the SMILES symbol of molecules. HALO is the presence/absence of halogen in the structure of molecules. HARD is the combination of BOND, NOSP, and HALO in the structure of compounds. Cmax, Nmax, and O max show the maximum number of rings (the range 0–9), the maximum number of nitrogen atoms, and the maximum number of oxygen atoms in the molecular structure, respectively. In addition, C5 and C6 indicate the presence of five- and six-membered rings in the molecular structures, respectively. The CW(x) represents the correlation weight of a SMILES feature or an HSG invariant.

The following equation indicates the correlation between the sum of correlation weights (DCW) of the optimal descriptors and pIC_50_ of the compounds:

2 $$ {\text{pIC}}_{{{5}0}} = {\text{a}} + {\text{b}} \times {\text{DCW}}\left( {{\text{T}}*,{\text{ N}}*} \right) $$

a is the intercept point and b is the slope of the line obtained by the least-squares method. DCW (Descriptors of Correlation Weights) is the sum of correlation weights for the optimal descriptor derived from HSG and SMILES and calculated by Monte Carlo optimization. The T* and N* indicate the optimal threshold value and the number of Monte Carlo optimization cycles, respectively.

A flowchart of a Monte Carlo optimization cycle is presented by Sokolovic et al. [26]. At first cycle, the CW(x) of features is randomly generated and then optimized based on the proposed objective function. There are different objective functions to obtain a reliable QSAR model in CORAL software. TF0, TF1 are two objective functions that we used here to obtain correlation weights for attributes and compare the extracted models based on each of them [27, 28].

3 $${\text{TF}}_{0}={\text{R}}_{\text{TRN}}+{\text{R}}_{\text{iTRN}}-\left|{\text{R}}_{\text{TRN}}-{\text{R}}_{\text{iTRN}}\right|\times \text{c}$$

4 $$ {\text{TF}}_{1} = {\text{TF}}_{0} + {\text{IIC}} \times c^{\prime} $$

The R_ATRN_ and R_PTRN_ denote the correlation coefficients between the experimental and predicted pIC_50_ for the active training and passive training sets, respectively and, c and c’ represent empirical values which are generally constant.

The IIC_CAL_ for calibration (CAL) set is obtained according to the following equation:

5 $$\text{IIC}={\text{R}}_{\text{CAL}}\times \frac{\text{min}\left({}^{-}{\text{MAE}}_{\text{CAL}, }{{}^{+}\text{MAE}}_{\text{CAL},}\right)}{\text{min}\left({}^{-}{\text{MAE}}_{\text{CAL}, }{{}^{+}\text{MAE}}_{\text{CAL},}\right)}$$

The R_CAL_ indicates the correlation coefficient for the calibration set. MAE_CAL_ (Mean Absolute Error for calibration set) is calculated based on Eqs 6 to 8:

6 $$^{-}{\text{MAE}}_{\text{CAL}}=-\frac{1}{\text{N}}\sum_{\text{K}=1}^{\text{N}}\left|{\Delta }_{\text{K}}\right| {\Delta }_{\text{K}}< 0,{\text{N}}^{-}\text{is}\;\text{the}\;\text{number}\;\text{of}\; \Delta {\text{k}} < 0$$

7 $$^{+}{\text{MAE}}_{\text{CAL }}=+\frac{1}{\text{N}}\sum_{\text{K}=1}^{\text{N}}\left|{\Delta }_{\text{K}}\right| {\Delta }_{\text{K}}\ge 0,{\text{N}}^{+}\text{is}\;\text{the}\;\text{number}\;\text{of}\; \Delta {\text{k}}\ge 0$$

8 $${\Delta }_{\text{k}}={\text{Exerimental}}_{\text{k}}-{\text{predicted}}_{\text{k}}$$

The ‘k’ is the index (1, 2... N) and the experimental k and predicted k are related to the pIC_50_. The CWs for each attribute of Split 1 is provided as an example in Additional file 1: Table S1, total number of attributes is 383.

### QSAR model Validation

There are various criteria for evaluating the predictive ability of QSAR models, such as internal validation, external validation, and Y-scrambling. In this study, some standard statistical criteria were used to check the validity of the QSAR models, such as coefficient of determination (R^2^), concordance correlation coefficient (CCC), Q^2^, Q^2^_F1_, Q^2^_F2_, Q^2^_F3_, standard error of estimation (s), mean absolute error (MAE), r^2^_m_ and new Y-scrambling criteria (${\text{C}}_{{\text{R}}_{\text{P}}^{2}}$) [29–32]. In addition, the IIC of models was used to improve the predictability of the models [33, 34].

### Applicability domain

The range of compounds for which a QSAR model can make reliable predictions is defined based on the applicability domain (AD) of model as the Organization of Economic Co-operation and Development (OECD) principle 3. Here, the AD is calculated based on the distribution of SMILES features in the training and calibration sets and is defined as “${\text{Defect}}_{{\text{A}}_{\text{K}}}$”[17].

9 $${\text{Defect}}_{{\text{F}}_{\text{K}}}=\frac{\left|{\text{P}}_{\text{TRN}}{(\text{A}}_{\text{K}})-{\text{P}}_{\text{CAL}}{(\text{F}}_{\text{K}})\right|}{{\text{N}}_{\text{TRN}}{(\text{A}}_{\text{K}})+{\text{N}}_{\text{CAL}}{(\text{F}}_{\text{K}})}$$

where P_TRN(Fk)_ and _PCAL(Fk)_ represent the probabilities of kth feature (F_k_) in the training and calibration set, respectively; N_TRN(Fk)_ and N_CAL(Fk)_ denote the frequency of kth feature (F_k_) in the training and calibration set, respectively.

10 $${\text{Defect}}_{\text{Molecule}}=\sum_{\text{i}=1}^{{\text{F}}_{\text{K}}}{\text{Defect}}_{{\text{F}}_{\text{K}}}$$

According to the SMILES of molecules, the molecule is included in AD if:

11 $${\text{Defect}}_{\text{Molecule}}<2\times {\overline{\text{Defect}} }_{\text{TRN}}$$

where ${\overline{\text{Defect}} }_{\text{TRN}}$ is the average ${\text{Defect}}_{\text{molecule}}$ in the training set.

### The interpretation of QSAR models

CORAL software provides a simple approach to interpret QSAR models. Three categories of features can be extracted with numerical data of correlation weights in several Monte Carlo optimization cycles: (I) features with a positive correlation weight in all runs that increase the endpoint; (II) features with a negative correlation weight in all runs that decrease the endpoint; and also (III) features with both negative and positive correlation weight in different optimization runs, these features have an undefined role and not be classified as an increasing/decreasing promoters of the endpoint [35].

### Molecular docking study

Molecular docking method as a common virtual screening technique can help to find the most favorable ligand binding mode in protein for computer-aided drug discovery [36–38]. The X-ray crystallographic structures of SARS-COV-2 3CL^pro^ were obtained from the Protein Data Bank (PDB: 6XHO) based on a good experimental resolution (1.45 Å), R-value free (0.239), and R-value work (0.211). The native ligand in active site of this protein was ethyl (4R)-4-({N-[(4-methoxy-1H-indol-2-yl)carbonyl]-L-leucyl}amino)-5-[(3S)-2-oxopyrrolidin-3-yl]pentanoate (Query on V34), thus we use this pdb code for molecular docking of indole derivatives. The selected receptor for molecular docking simulation was the x-ray structure of SARS-COV-1 (PDB ID: 1UK4) based on a good experimental resolution (2.5 Å), R-value free (0.231), and R-value work (0.213). The native ligand in active site of this protein was 5-mer peptide. 6XHO and1 UK4 structures consist of a dimer composed of two identical sequences. The side chain A was chosen for molecular docking and the side chain B was removed. The protein structure was prepared using adding hydrogens removing water molecules and native ligands. Then, the Kollmann charges were assigned to the receptor. All compounds were sketched using the by ChemOffice15 (PerkinElmer Inc.), and assigned gasteiger charges and energy optimization of ligands using the steepest descent algorithm carried out by Open Babel [39]. The docking studies were done with the Smina program. Smina is a version of AutoDock Vina with a modified scoring function that is particularly optimized to offer high-throughput scoring (http://smina.sf.net) [40].

The grid parameter file is according to the grid box that comprised 20 × 20 × 20 points with 1 Å space and was centered on the active site of SARS-COV-2 3CL^pro^ (x = 9.412, y = 1.383, and z = 8.836). The grid parameter file is according to the grid box that comprised 14 × 14 × 14 points with 1 Å space and was centered on the active site of SARS-COV-1 (x = 66.036, y = 3.288, and z = 5.254).

The X-ray crystallographic structures of SARS-COV-1, SARS-COV-2 3CL^pro^ were obtained from the Protein Data Bank (PDB: 1UK and 6XHO). The structures of compounds were drawn by BIOVIA Discovery Studio Visualizer 2021. The calculation of energy optimization was done using the steepest descent method. Smina was performed with default settings for three proteins and 9 best conformations of ligand were introduced (Additional file 1: Table S4). The computational docking approach was evaluated based on the root-mean-square deviation (RMSD) value from re-docking the co-crystalized native ligand back into the active pocket site of the receptor [41].

## Results and discussion

### QSAR models

To build the reliable QSAR models, two objective functions were used: objective function without IIC (TF0) and with IIC (TF1). The range of finding the optimal threshold value (T) and the number of epochs (N) were 1–3 and 1–15, respectively. The QSAR models to predict the inhibitory activity against SARS 3CL^pro^ for four splits were built based on TF1 are given below:

Split 1:

12 $${\text{pIC}}{50}=2.4816 \left(\pm 0.0328\right)+0.0572 \left(\pm 0.0005\right)\times \text{DCW}\left(\text{1,14}\right)$$

$${\text{R}}_{\text{ATRN}}^{2}=0.94{,\text{ n}}_{\text{TRN}}=25; {\text{R}}_{\text{PTRN}}^{2}=0.95{,\text{ n}}_{\text{PTRN}}=20;{\text{ R}}_{\text{CAL}}^{2}=0.92{,\text{ n}}_{\text{CAL}}=16;{\text{ R}}_{\text{VAL}}^{2}=0.88{,\text{ n}}_{\text{VAL}}=20$$

Split 2:

13 $${\text{pIC}}{50}=-0.0804 (\pm 0.0679)+0.0972 (\pm 0.0010)\times \text{DCW}(\text{1,12})$$

$${\text{R}}_{\text{ATRN}}^{2}=0.94{,\text{ n}}_{\text{ATRN}}=24; {\text{R}}_{\text{PTRN}}^{2}=0.94{,\text{ n}}_{\text{PTRN}}=19;{\text{ R}}_{\text{CAL}}^{2}=0.90{,\text{ n}}_{\text{CAL}}=16;{\text{ R}}_{\text{VAL}}^{2}=0.83{,\text{ n}}_{\text{VAL}}=22$$

Split 3:

14 $${\text{pIC}}{50}=-0.1674 \left(\pm 0.0477\right)+0.1226 \left(\pm 0.0010\right)\times \text{DCW}\left(\text{1,6}\right)$$

$${\text{R}}_{\text{ATRN}}^{2}=0.96{,\text{ n}}_{\text{ATRN}}=23; {\text{R}}_{\text{PTRN}}^{2}=0.93{,\text{ n}}_{\text{PTRN}}=20;{\text{ R}}_{\text{CAL}}^{2}=0.87{,\text{ n}}_{\text{CAL}}=16;{\text{ R}}_{\text{VAL}}^{2}=0.92{,\text{ n}}_{\text{VAL}}=22$$

Split 4:

15 $${\text{pIC}}{50}=0.3203 (\pm 0.0545)+0.1004 (\pm 0.0011)\times \text{DCW}(\text{1,10})$$

$${\text{R}}_{\text{ATRN}}^{2}=0.96{,\text{ n}}_{\text{ATRN}}=24; {\text{R}}_{\text{PTRN}}^{2}=0.96{,\text{ n}}_{\text{PTRN}}=21;{\text{ R}}_{\text{CAL}}^{2}=0.88{,\text{ n}}_{\text{CAL}}=16; {\text{R}}_{\text{VAL}}^{2}=0.81{,\text{ n}}_{\text{VAL}}=20$$

where ${\text{R}}_{\text{ATRN}}^{2}$, ${\text{R}}_{\text{PTRN}}^{2}$ R^2^_CAL_, and R^2^_VAL_ are coefficient of determination for active training, passive training, calibration, and validation set, respectively. ${\text{ n}}_{\text{ATRN}}, {{\text{ n}}_{\text{PTRN}},\text{ n}}_{\text{CAL}}$, and ${\text{n}}_{\text{VAL}}$ indicate the number of molecules in the training, calibration, and validation set, respectively.

Table 2 indicates the statistical criteria of QSAR models for predicting of pIC_50_ isatin and indole derivatives based on TF0 and TF1 for each split. Regarding the QSAR models, the models developed based on IIC (TF1) are more predictive than the models developed using TF1. Therefore, it can be stated that the QSAR models built with the modified objective function TF1 using IIC are more reliable and robust than the models built by the objective function TF0. Thus, the QSAR model built for split 3 with TF1 was selected as the best model because the coefficient of determination (R^2^) was the highest for the validation set of this model.

**Table 2:** Statistical parameters of QSAR models for prediction of pIC_50_

Split	Target function	Set	n	R^2^	CCC	IIC	Q^2^	${\text{Q}}_{{\text{F}}_{1}}^{2}$	${\text{Q}}_{{\text{F}}_{2}}^{2}$	${\text{Q}}_{{\text{F}}_{3}}^{2}$	s	MAE	${r}_{m}^{2}$	${\text{C}}_{{\text{R}}_{\text{P}}^{2}}$
1	TF0	ATRN	25	0.9992	0.9996	0.9225	0.9990				0.030	0.017		0.9663
		PTRN	20	0.9991	0.9855	0.5687	0.9989				0.195	0.159		0.9761
		CAL	16	0.7308	0.8293	0.6755	0.6265	0.6274	0.6027	0.5712	0.762	0.571	0.6807	0.6995
		VAL	20	0.6200	0.7652	0.5845	0.5353				0.7476	0.5390	0.6173	
	TF1	ATRN	25	0.9419	0.9701	0.6470	0.9330				0.253	0.211		0.9317
		PTRN	20	0.9470	0.9322	0.5838	0.9343				0.414	0.342		0.9174
		CAL	16	0.9229	0.9173	0.9606	0.9015	0.8788	0.8708	0.8605	0.435	0.364	0.5968	0.9043
		VAL	20	0.8804	0.9235	0.8546	0.8603				0.3770	0.3123	0.8545	
2	TF0	ATRN	24	0.9995	0.9997	0.8459	0.9994				0.026	0.018		0.9767
		PTRN	19	0.9995	0.9678	0.9998	0.9994				0.299	0.262		0.9646
		CAL	16	0.6102	0.6710	0.2277	0.5350	0.3128	0.2694	0.4007	0.908	0.656	0.3894	0.5836
		VAL	22	0.7387	0.8535	0.7343	0.6710				0.6126	0.4913	0.7166	
	TF1	ATRN	24	0.9407	0.9694	0.6928	0.9300				0.280	0.225		0.9214
		PTRN	19	0.9405	0.9245	0.2175	0.9192				0.423	0.334		0.9240
		CAL	16	0.9044	0.9487	0.9509	0.8773	0.9090	0.9033	0.9206	0.330	0.260	0.7991	0.8963
		VAL	22	0.8258	0.9055	0.6769	0.7964				0.4563	0.3460	0.7153	
3	TF0	ATRN	23	0.9995	0.9998	0.9167	0.9994				0.024	0.016		0.9861
		PTRN	20	0.9979	0.9847	0.9990	0.9976				0.209	0.193		0.9506
		CAL	16	0.7578	0.8256	0.7414	0.6978	0.5360	0.5276	0.6404	0.705	0.546	0.6655	0.7177
		VAL	22	0.7342	0.7536	0.4182	0.6885				1.0149	0.7729	0.5618	
	TF1	ATRN	23	0.9581	0.9786	0.7529	0.9514				0.221	0.170		0.9430
		PTRN	20	0.9283	0.9419	0.9607	0.9131				0.383	0.316		0.8977
		CAL	16	0.8668	0.9126	0.9310	0.8226	0.7897	0.7858	0.8370	0.475	0.361	0.7721	0.8214
		VAL	22	0.9170	0.9172	0.4932	0.8975				0.5134	0.3963	0.7490	
4	TF0	ATRN	24	0.9992	0.9996	0.5998	0.9990				0.030	0.019		0.9765
		PTRN	21	0.9990	0.9679	0.3260	0.9988				0.265	0.193		0.9816
		CAL	16	0.6548	0.7855	0.5333	0.5865	0.6178	0.5361	0.5700	0.747	0.605	0.5882	0.6127
		VAL	20	0.6223	0.7862	0.4861	0.5550				0.7126	0.4863	0.5345	
	TF1	ATRN	24	0.9580	0.9786	0.8282	0.9494				0.217	0.168		0.9414
		PTRN	21	0.9569	0.9651	0.4967	0.9447				0.292	0.227		0.9414
		CAL	16	0.8786	0.9322	0.9373	0.8404	0.8795	0.8538	0.8644	0.420	0.343	0.8631	0.8476
		VAL	20	0.8090	0.8887	0.3711	0.7787				0.5356	0.3886	0.7850	

Y-randomization test (Y-test) was done by CORAL software to confirm the non-chance correlation of developed QSAR models. After ten repetitions of new random models were developed and the values of average value of R^2^ were found below 0.1 (see Additional file 1: Table S2). These values confirm that the correlation between pIC_50_ and molecular attributes is not based on chance correlation. Moreover, for the Y-randomization test, the value of CR^2^p for all models was more than 0.8 (Table 2).

Additional file 1: Table S3 shows the SMILES symbol of isatin and indole derivatives, the set of each compound, the observed and calculated pIC_50_ of four models, and AD in four splits using TF1. The average ${\overline{\text{Defect}} }_{\text{TRN}}$ for Split 1 to 4 of constructed models based of TF0 are 5.91, 3.19, 5.18, and 5.05, respectively. So, compounds fall into AD if DefectSMILES < 11.82, 6.38, 10.36, and 10.10, for split 1 to 4 respectively. The percentages of data set in the AD of models were 82, 82, 83, and 88 for splits 1–4, respectively. This revealed that the four prediction models were capable of predicting more than 80% of the new data (Additional file 1: Table S3).

**Fig. 1:**
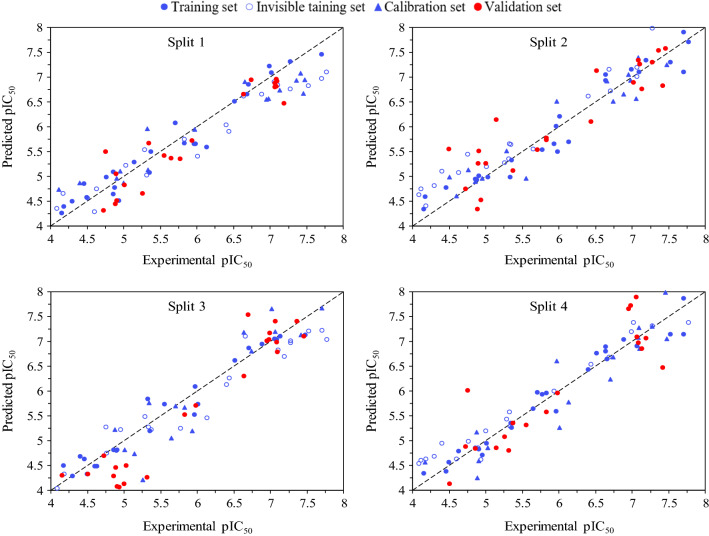
The graphical representation of the observed versus prediccted pIC_50_ for split 1 to 4

Figure 1 displays the plots of the calculated versus observed pIC_50_ of SARS 3CL^pro^ inhibitors for four models developed based on TF1. It also shows that there is good agreement between the observed and experimental pIC_50_.

### Mechanistic interpretation

Mechanistic interpretation as the fifth OECD principle of QSAR modeling states that the molecular features responsible for increased or decreased activity should be investigated whenever possible. The interpretation of the model can help to design and identify new isatin- and indole-based derivatives. The list of structural features extracted from the best QSAR model (split 3) for three independent probes is shown in Table 3. A short description of these descriptors is presented in the comments column of Table 3 which shows the structural features of increasing or decreasing pIC_50_ of isatin and indole derivatives. The identified promotors in the increase of pIC_50_ include the presence of nitrogen with double bond, presence of nitrogen with oxygen, presence of oxygen with double bond, presence of at least one ring, combination of aliphatic oxygen with double bond, presence of oxygen with double bond and branching and presence of aromatic carbon in first ring. The promoters of decrease of SARS 3CL^pro^ inhibitory activity of isatin and indole derivatives are the presence of nitrogen with sulfur, presence of consecutive aliphatic carbon with aliphatic nitrogen with branching, presence of aromatic carbon with branching in fourth ring and presence of aliphatic carbon with branching in fourth ring.

**Table 3:** The list of structural attributes increases or decrease the pIC_50_ of isatin and indole derivatives based on the Split 3 model for three independent probes

SA_K_	Cws Probe 1	CWs Probe 2	CWs Probe 3	NSs	NSc	NSv	Defect [SA_k_]	Comments
+ + + + N–-B2 = =	0.81977	1.44792	3.3629	23	20	16	0	Presence of nitrogen with double bond
+ + + + N–-O = = =	0.55248	1.60119	0.9952	23	20	16	0	Presence of nitrogen with oxygen
+ + + + O–-B2 = =	3.11773	2.3733	4.25607	23	20	16	0	Presence of oxygen with double bond
1……….	2.69283	0.10613	3.13178	23	20	16	0	Presence of at least one ring
O…(……	0.4556	0.00742	0.17344	23	20	16	0	Combination of aliphatic oxygen with branching
O… = ……	0.19142	0.11911	0.49273	23	20	16	0	Combination of aliphatic oxygen with double bond
3……….	0.32927	0.44463	2.31771	22	19	16	0.0011	Presence of at least three rings
= …(……	0.1846	0.14941	0.0791	22	20	16	0.0011	Combination of double bound with branching
O… = …(…	0.15551	0.31959	0.3984	22	20	16	0.0011	Presence of oxygen with double bond and branching
c…2……	1.17117	1.05998	0.02425	22	19	16	0.0011	Presence of aromatic carbon in second ring
c…c…2…	0.18057	0.67353	0.16569	22	19	16	0.0011	Presence of two consecutive aromatic carbon in second ring
N…(……	0.3027	0.16119	0.46805	19	15	14	0.0015	Combination of nitrogen with branching
BOND10000000	0.33172	0.06765	0.32395	18	12	7	0.0138	Presence of double bounds
C…(… = …	1.0509	0.49087	0.32091	16	20	15	0.0078	Presence of aliphatic carbon with branching and double bond
c…1……	0.04992	2.36042	0.34589	14	14	12	0.0054	Presence of aromatic carbon in first ring
+ + + + N–-S = = =	− 0.60102	− 0.03774	− 0.7567	13	10	8	0.0031	Presence of nitrogen with sulfur
N…1……	− 1.44414	− 2.57584	− 0.89628	7	2	4	0.0082	Presence of aliphatic nitrogen in firth ring
C…N…(…	− 0.13702	− 1.66665	− 1.60758	8	8	6	0.0054	Presence of consecutive aliphatic carbon with aliphatic nitrogen with branching
4…c…(…	− 0.85897	− 0.24461	− 0.27774	6	6	5	0.0047	Presence of aromatic carbon with branching in fourth ring
N…3…C…	− 0.85012	− 0.27675	− 0.27575	4	0	1	0.0223	Presence of aliphatic nitrogen and carbon in third ring
C…(…4…	− 0.7243	− 0.07997	− 0.11114	3	5	0	1	Presence of aliphatic carbon with branching in fourth ring
C5…AH.2…	− 0.36619	− 0.87409	− 0.56113	3	4	4	0.0171	Presence of two five-member rings with aromaticity and heteroatoms
s…4……	− 0.58556	− 0.70714	− 0.26015	3	3	3	0.0095	Presence of aromatic sulphur in the fourth ring
+ + + + I–-N = = =	− 0.95359	− 1.09861	− 0.30426	2	2	1	0.0082	Presence of iodine with nitrogen
C…c…2…	− 0.84725	− 0.3365	− 0.29322	2	0	1	0.0082	Prsence of consecutive aliphatic carbon with aromatic carbon in second ring
[…C…@…	− 0.26604	− 1.1151	− 0.15705	2	3	3	0.0201	Presence of aliphatic carbon with stereo-chemical (3D) bond

Based on the favorable structural features and using the most active molecules among the 81 inhibitors which were gathered from literature, some compounds synthesized in various studies were extracted from ChEMBL database. In the ChEMBL database, newly synthesized compounds can be extracted with percentage similarity with desired compound, so we entered the ligand with the highest activity into ChEMBL and extracted some similar compounds from this database. The inhibitory activity (pIC_50_) of selected structures was calculated using best QSAR model (Split 3). Finally, eight most active compounds (isatin and indole scaffolds with most pIC_50_) were selected and introduced which are listed in Table 4. The predicted pIC_50_ range for the extracted compounds based on average prediction of four models was between 7.35 and 8.30. The AD analysis of these compounds based on the Split 3 model (the best model) shows that they fall into AD except for CHEMBL3103276.

**Table 4:** The average predicted pIC_50_, IC_50_, affinity, based on four models for eight extracted compounds from CHEMBL data search

Structure	pIC_50_	IC_50_ (µM)	Affinity (Kcal mol^−1^)	PDB	Structure	pIC_50_	IC_50_ (µM)	Affinity (Kcal mol^−1^)	PDB
CHEMBL4524939 Indole scaffold	7.99	0.010	-9.7	6XHO	CHEMBL4443007 Indole scaffold	8.30	0.005	-10.1	6XHO
CHEMBL4458417 Indole scaffold	7.36	0.043	-9.6	6XHO	CHEMBL383761 Isatin scaffold	7.69	0.020	-9.1	1UK4
CHEMBL4452760 Indole scaffold	8.11	0.008	-10.1	6XHO	CHEMBL210543 Isatin scaffold	7.99	0.010	-9.4	1UK4
CHEMBL4565907 Indole scaffold	7.6	0.025	-9.7	6XHO	CHEMBL3103276 Isatin scaffold	7.89	0.013	-9.4	1UK4

### Molecular docking analysis

**Fig. 2:**
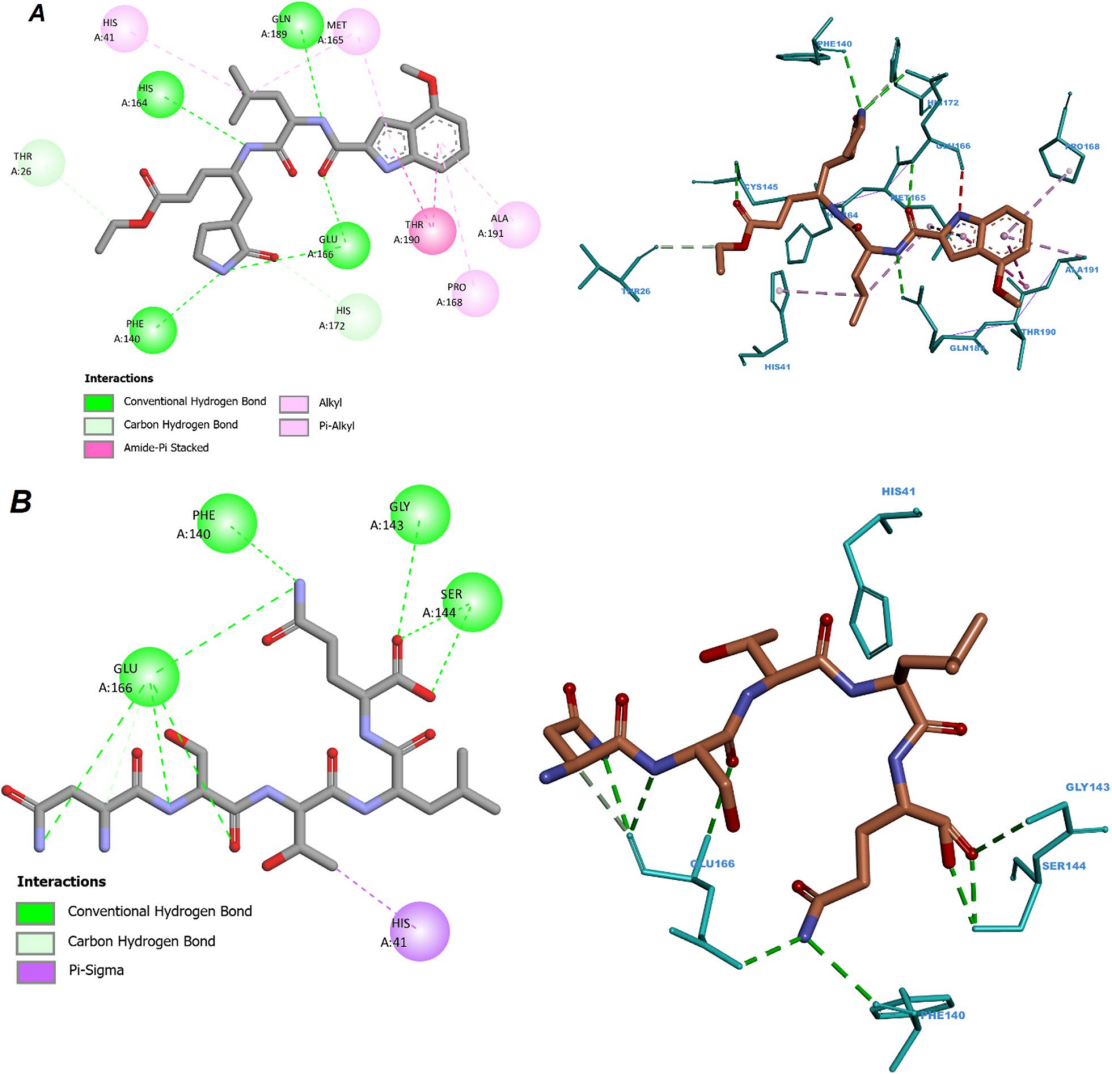
V34 interaction patterns with active residues in the SARS-COV-2 3CLpro pocket (A), 5-mer peptide interaction patterns with active residues in the SARS-COV-1 pocket (B)

First, we perform a re-docking of the V34 ligand with the SARS-COV-2 3CL^pro^ and 5-mer peptide with SARS-COV-1 receptors; this is done to validate the molecular docking protocol and also to get insight into the reference active amino acid residues involved in interactions inside the SARS-COV-2 3CL^pro^ and SARS-COV-1 protein pocket (PDB code: 6XHO and 1UK4). Figure 2 displays 3D and 2D visualizations of the re-docking pathways of V34 inside the COVID-2 3CL^pro^ and 5-mer peptide inside the SARS-COV-1 protein pockets with − 8.07 and − 9.4 kcal/mol, respectively. Figures indicate that the re-dock V34 located in the active site of SARS-COV-2 3CL^pro^ interacts with the THR26, HIS41, PHE140, CYS145, HIS164, MET165, GLU166, PRO168, HIS172, GLN189, THR190, and ALA191. Also, the re-dock 5-mer peptide located in the active site of SARS-COV-1 interacts with the HIS41, PHE140, GLY143, SER144, and GLU166. These interactions were hydrophobic and hydrogen bonds. The root-mean-square deviation (RMSD) values were 0.14 and 1.1 Å for native and re-docked ligands of V34 and 5-mer peptide, respectively; which are lower than the tolerable marginal value of 2 Å (Additional file 1: Fig. S1).

**Fig. 3:**
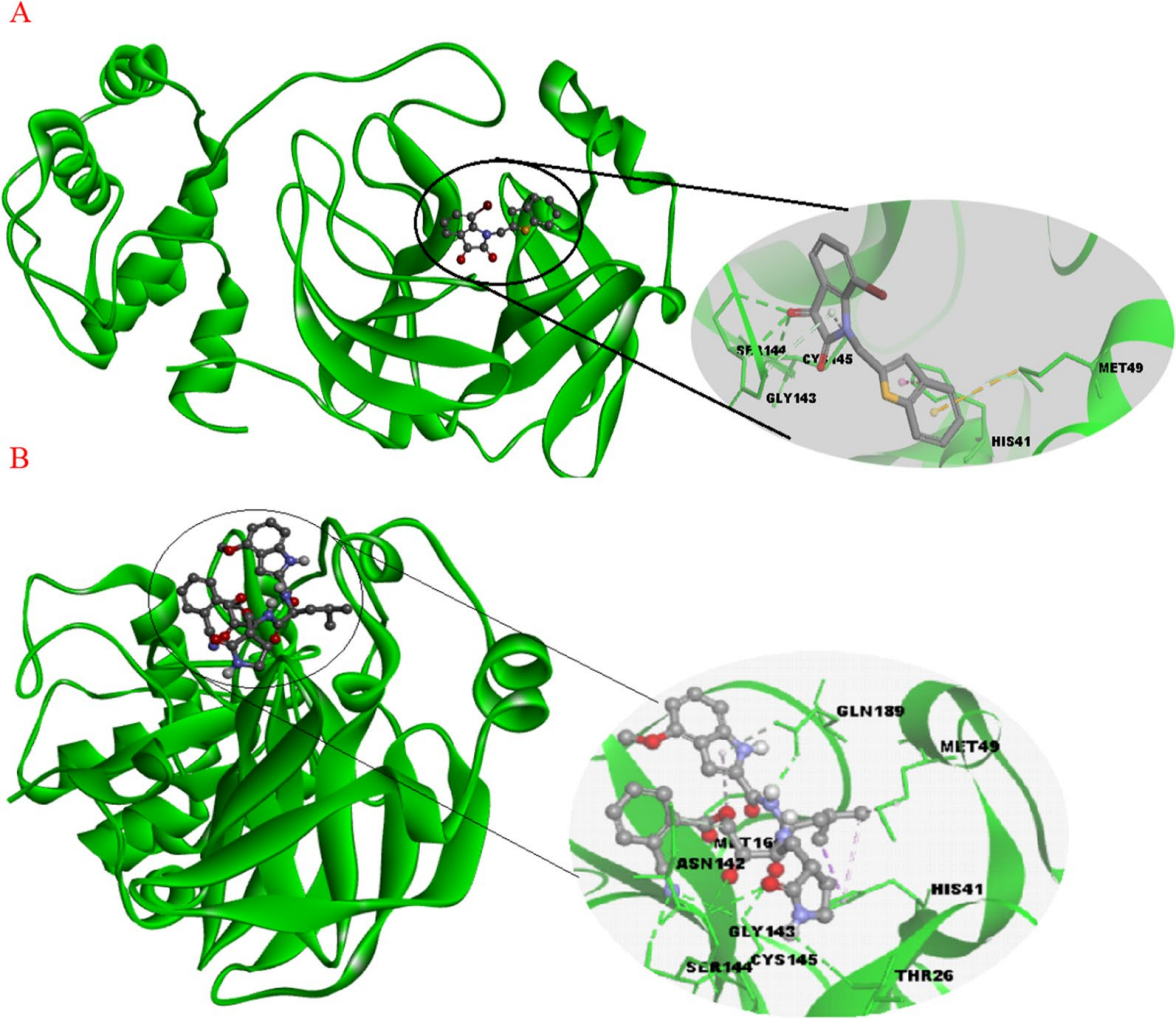
Three-dimensional diagram of compound 12(**A**) and 53(**B**) into the binding pocket of SARS-COV-1 3CL^p^

**Fig. 4:**
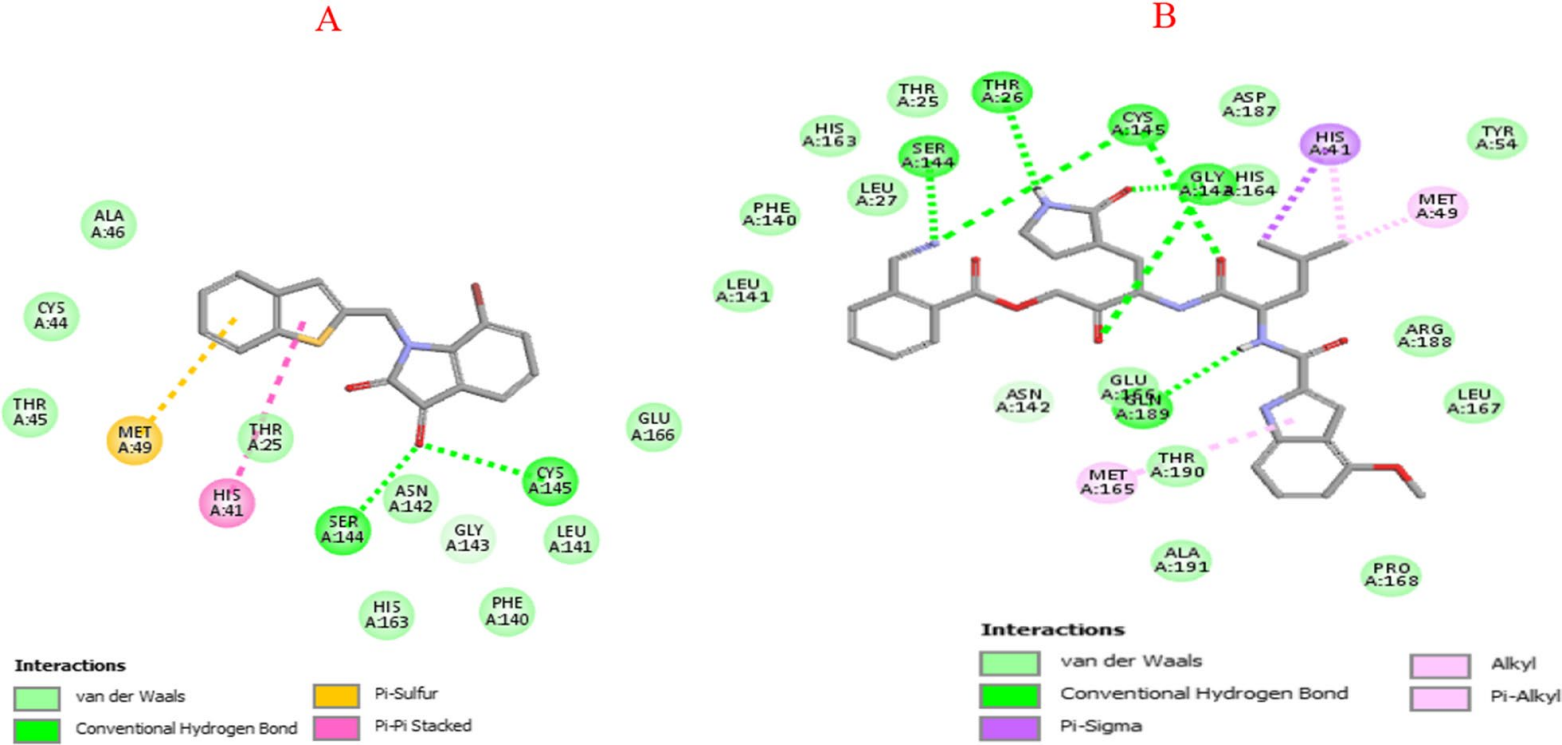
Two‐dimensional diagram of compound 12 (**A**) and 53 (**B**) interactions with binding site residues of SARS-COV-1 3CL^pro^

Figure 3a and b shows that the compound 12 and 53 were placed into the binding pocket of SARS-COV-1 3CL^pro^ by representing three-dimensional diagram. Two‐dimensional diagram of compound 12 and 53 interactions was presented in Fig. 4a and b the compounds formed some important interactions with binding site residues of SARS-COV-1 3CLpro. As the molecular docking results are shown in Fig. 3a, the compound 12 formed two hydrogen bond interactions with SER144 and CYS145 the binding site of SARS-COV-1 3CL^pro^. Also, it has two hydrophobic interactions with HIS41 and MET49. Moreover, ALA46, CYS44, THR45, THR25, ASN142, GLY143, HIS163, PHE140, LEU141 and GLU166 have van der Walls interaction with the protein. Figure 3b shows various interactions of compound 57 with HIS41, MET49 and MET165, along with some hydrophobic interactions. In addition, the complex formed hydrogen bond interactions with residues SER144, THR26, CYS145, GLY143 and GLN189. LEU141, PHE140, HIS163, LEU27, THR25, ASN142, GLU166, THR190, ALA191, TYR54, ARG188, LEU167 and PRO168 had van der Walls interaction with the protein.

Comparing the molecular docking results of re-docked native ligands and compounds 12 and 53 as the most activist compounds; we can notice that all compounds 12 and 53 interacted with the majority of active residues in the COV-2 3CLpro and SARS-COV-1 pockets with which native ligands interacted.

Molecular docking results agree with some promoters regarding the increase in pIC_50_ in QSAR models; for instance, compounds 12 and 53 contain oxygen with double bonds, at least one ring, and branching, all of which interact with amino acids residues in protein active sites via hydrogen bonds and hydrophobic interactions.

Hexachlorophene was used as a SARS 3CL^pro^ standard inhibitor (IC_50_ = 5 µM) according to Liu et al. [42]. We docked Hexachlorophene into the active site of 6XHO. The best binding mode of the Hexachlorophene in the binding site of SARS-COV-1 3CL^pro^ (pdb: 6XHO) was − 8.05 kcal/mol.

Eight extracted compounds from CHEMBL based on scaffold of isatin or indole were docked into 1UK4 and 6XHO as well. Two and three‐dimensional diagrams of the interaction of the eight ligands from CHEMBLE with their receptors are presented in Additional file 1: Fig. S2. Molecular docking analysis shows that these ligands with the majority of active residues in the COV-2 3CLpro and SARS-COV-1 pockets with which native ligands interacted. As before we mentioned it for the activist compounds 12 and 53. It confirmed that indole and isatin are important cores in interaction with targets. As can be seen in Table 4, all eight compounds had higher binding energy compared to the most active compounds in data set and hexachlorophene. The results present a very good correlation between results obtained from Monte Carlo optimization modeling and molecular docking studies.

## ADMET results

In silico ADMET (absorption, distribution, metabolism, excretion, and toxicity) screening of compounds can reduce the cost and time associated with the in vitro assay and/or in vivo experiments [43]. AdmetSAR online database was used to predict ADMET properties of extracted isatin- and indole-based compounds [44]. As ADMET properties are shown in Table 5, all eight compounds showed positive results for human intestinal absorption. Furthermore, it is necessary to check whether the proposed molecules are non-toxic because it plays an important role in the selection of drugs. Ames test was negative for all compounds except CHEMBL4443007 and based on acute oral toxicity all compounds were classified as non-toxic.

**Table 5:** ADMET prediction for eight extracted compounds from CHEMBL

Compound	Human intestinal absorption	ClogP	Ames test	Acute oral toxicity	Drug likeness	Drug score
CHEMBL4524939	+ (0.9816)	5.36	No	III	− 2.47	0.16
CHEMBL4458417	+ (0.9816)	4.02	No	III	6.12	0.56
CHEMBL4452760	+ (0.9792)	3.85	No	III	1.76	0.46
CHEMBL4565907	+ (0.9774)	4.00	No	III	2.59	0.51
CHEMBL4443007	+ (0.9816)	4.05	Yes	III	6.75	0.48
CHEMBL383761	+ (0.9670)	2.67	No	III	− 3.73	0.34
CHEMBL210543	+ (0.9914)	3.10	No	III	− 4.66	0.32
CHEMBL3103276	+ (0.9761)	3.43	No	III	− 5.60	0.17

The Osiris Property Explorer (OPE) tool was used to assess the fragment-based drug-likeness of the extracted compounds [45, 46]. A positive value (0.1–10) indicates that the compound mainly contains fragments that are often found in commercial drugs. Also, using this program, the overall drug scores were evaluated that combines drug-likeness, ClogP, ClogS, molecular weight, and toxicity risk factors in one single value where the frequency of occurrence of each fragment is determined within the collection of approved drugs and within Fluka non-medicinal chemicals.

Finally, based on the results of the OPE study, compounds CHEMBL4458417 and CHEMBL4565907 both containing an indole scaffold with the positive values of drug-likeness and the highest drug-score can be introduced as selected leads.

## Conclusion

Four simple, predictive, and reliable QSAR models were developed for the pIC_50_ values of 81 isatin and indole derivatives that inhibit SARS 3CL^pro^ using Monte Carlo with the index of ideality of correlation (IIC) as the objective function. The statistical parameters of the models were suitable with high predictive power (${R}_{Val}^{2}$ = 0.81–0.92, and MAE = 0.31–0.40). The four proposed models were satisfactory for predicting new isatin and indole derivatives as candidates for SARS 3CL^pro^ inhibitors and can be used for pre-synthesis evaluation of new isatin and indole derivatives. A mechanistic interpretation of the models was done by examining the correlation weights of the different extracted molecular features extracted in several Monte Carlo optimization runs. These features were used to extract eight new and more active isatin and indole derivatives from the ChEMBL database. The activity of new compounds was further verified by molecular docking studies. The activity of the new compounds was further confirmed by molecular docking studies. The binding energy of these molecules with residues of active site were in correlation with calculated pIC_50_. Finally, the compounds CHEMBL4458417 and CHEMBL4565907 both containing an indole scaffold with the positive values of drug-likeness and the highest drug-score were introduced as selected leads.

## Supplementary Information

**Additional file1: Table S1.** CWs for each attribute of Split 1. **Table S2. **The results of Y-randomization test for all splits constructed based on TF1. **Table S3. **SMILES notations of isatin and indole derivatives, the compound set, their experimental, predicted pIC_50_, and applicability domain in four splits using TF1. **Table S4.** The affinity of nine conformations docked into SARS-COV-1 3CLpro (PDB: 1UK4 and 6XHO) for compounds 12 and 53. **Figure S1**. 3D superposition of original (black) and re-docked (yellow) (A) V34 ligand in the 6XHO (RMSD=0.14 Å), (A) 5-mer peptide ligand in the 1UK4 (RMSD=1.1 Å). **Figure S2.** Two and three‐dimensional diagram of (A) CHEMBL4524939 (B) CHEMBL4458417 (C) CHEMBL4452760 (D) CHEMBL4565907 (E) CHEMBL4443007 interactions with binding site residues of SARS-COV-1 3CLpro (6XHO) and (F) CHEMBL383761 (G) CHEMBL210543 (H) CHEMBL3103276interactions with binding site residues of SARS-COV-1 (1UK4).

## Data Availability

The datasets used and/or analyzed during the current study are available from the corresponding author on reasonable request.
