# Supplementary material for: QSAR and molecular docking studies of isatin and indole derivatives as SARS 3CLpro inhibitors
Source: BMC Chem. 2023 Apr 7;17(1):32. doi: 10.1186/s13065-023-00947-w (PMC10079496; doi:10.1186/s13065-023-00947-w)
Supplement: Supplementary file 1 — Additional file1: Table S1. CWs for each attribute of Split 1. Table S2. The results of Y-randomization test for all splits constructed based on TF1. Table S3. SMILES notations of isatin and indole derivatives, the compound set, their experimental, predicted pIC50, and applicability domain in four splits using TF1. Table S4. The affinity of nine conformations docked into SARS-COV-1 3CLpro (PDB: 1UK4 and 6XHO) for compounds 12 and 53. Figure S1. 3D superposition of original (black) and re-docked (yellow) (A) V34 ligand in the 6XHO (RMSD=0.14 Å), (A) 5-mer peptide ligand in the 1UK4 (RMSD=1.1 Å). Figure S2. Two and three‐dimensional diagram of (A) CHEMBL4524939 (B) CHEMBL4458417 (C) CHEMBL4452760 (D) CHEMBL4565907 (E) CHEMBL4443007 interactions with binding site residues of SARS-COV-1 3CLpro (6XHO) and (F) CHEMBL383761 (G) CHEMBL210543 (H) CHEMBL3103276interactions with binding site residues of SARS-COV-1 (1UK4). [file 13065_2023_947_MOESM1_ESM.docx]

**Table S1.** CWs for each attribute of Split 1.

| ID | Descriptor | CW(x) | ID | Descriptor | CW(x) | ID | Descriptor | CW(x) |
| --- | --- | --- | --- | --- | --- | --- | --- | --- |
| 1 | N...2....... | -5.39244 | 129 | C...2...=... | -0.04025 | 257 | [...H...@... | 0.40579 |
| 2 | C...N...1... | -3.90792 | 130 | C6...AH.5... | -0.03764 | 258 | c...c...(... | 0.4065 |
| 3 | C...3...(... | -3.02444 | 131 | c...1...O... | -0.0337 | 259 | c...c...4... | 0.40738 |
| 4 | =...4....... | -2.52504 | 132 | n...[...3... | -0.02433 | 260 | H...[...1... | 0.40777 |
| 5 | s...5...c... | -2.34503 | 133 | [...O....... | -0.02398 | 261 | [...C...@@.. | 0.4163 |
| 6 | N...1...C... | -2.26176 | 134 | n...1...n... | -0.02322 | 262 | 2...C...(... | 0.42186 |
| 7 | n...c...5... | -2.14522 | 135 | O........... | -0.02074 | 263 | HALO01000000 | 0.42816 |
| 8 | ++++O---S=== | -2.04408 | 136 | Omax.6...... | -0.01997 | 264 | C...O...(... | 0.43172 |
| 9 | s........... | -1.66388 | 137 | c...(...2... | -0.0055 | 265 | c...3...N... | 0.43386 |
| 10 | N...1....... | -1.6391 | 138 | c...Cl...... | -0.00164 | 266 | O...c...1... | 0.43951 |
| 11 | N...C...4... | -1.57329 | 139 | C...(...=... | 0.00652 | 267 | n...O....... | 0.44206 |
| 12 | C...N...4... | -1.55189 | 140 | C...C...5... | 0.00901 | 268 | N...#....... | 0.4427 |
| 13 | c...O....... | -1.34636 | 141 | N...C....... | 0.02305 | 269 | c...3...(... | 0.44406 |
| 14 | $10011000000 | -1.24216 | 142 | O...(....... | 0.02567 | 270 | c...c...2... | 0.44562 |
| 15 | c...(...I... | -1.17249 | 143 | n...c...c... | 0.03107 | 271 | [...n...H... | 0.45073 |
| 16 | c...2...(... | -1.17102 | 144 | c...c...1... | 0.03186 | 272 | O...=...3... | 0.45847 |
| 17 | o...n....... | -1.14974 | 145 | N...C...1... | 0.03435 | 273 | C...O...C... | 0.45918 |
| 18 | Omax.7...... | -1.11478 | 146 | c...(....... | 0.04001 | 274 | Omax.5...... | 0.46107 |
| 19 | 4...c...(... | -1.09804 | 147 | ++++I---O=== | 0.04072 | 275 | N...C...(... | 0.46762 |
| 20 | C...c...2... | -1.09378 | 148 | ++++N---B3== | 0.04077 | 276 | 1...c...(... | 0.47613 |
| 21 | N...5....... | -1.06028 | 149 | =...1....... | 0.04723 | 277 | $10111000000 | 0.48978 |
| 22 | n...c...(... | -1.05325 | 150 | C...3....... | 0.04777 | 278 | n...1....... | 0.49076 |
| 23 | Nmax.3...... | -1.02939 | 151 | O...4....... | 0.04839 | 279 | n...[...2... | 0.49081 |
| 24 | c...n...4... | -1.01496 | 152 | c...c...[... | 0.04854 | 280 | 3...C...1... | 0.49115 |
| 25 | O...(...(... | -0.99604 | 153 | C6...A..2... | 0.04923 | 281 | N...C...2... | 0.50383 |
| 26 | c...c...c... | -0.98096 | 154 | (........... | 0.04947 | 282 | H........... | 0.53271 |
| 27 | C...(....... | -0.95043 | 155 | Cmax.3...... | 0.05038 | 283 | [...(...O... | 0.54261 |
| 28 | 3...N...(... | -0.935 | 156 | ++++CL--N=== | 0.05178 | 284 | ++++O---B2== | 0.54536 |
| 29 | 4...(....... | -0.91439 | 157 | Omax.9...... | 0.05886 | 285 | C...C...1... | 0.54648 |
| 30 | C...1....... | -0.91012 | 158 | C...N...(... | 0.06439 | 286 | O...n...1... | 0.56803 |
| 31 | O...=...4... | -0.90446 | 159 | (...(....... | 0.06463 | 287 | =...(...(... | 0.573 |
| 32 | S...(...2... | -0.88204 | 160 | O...(...O... | 0.0692 | 288 | S...(....... | 0.57609 |
| 33 | c...5....... | -0.86903 | 161 | [...2....... | 0.07282 | 289 | c...2...C... | 0.593 |
| 34 | C...[...C... | -0.8323 | 162 | c...1...c... | 0.07701 | 290 | c...2....... | 0.60528 |
| 35 | [...3...C... | -0.82568 | 163 | n...[...4... | 0.07757 | 291 | N...5...C... | 0.65204 |
| 36 | I...(....... | -0.80691 | 164 | 4...N...(... | 0.07922 | 292 | C...C...3... | 0.68335 |
| 37 | S...(...1... | -0.79298 | 165 | c...3...c... | 0.08535 | 293 | c...6...(... | 0.6865 |
| 38 | C...c...1... | -0.79191 | 166 | C...N...C... | 0.08781 | 294 | N...(....... | 0.69944 |
| 39 | N...3...C... | -0.7885 | 167 | n........... | 0.0919 | 295 | C...3...C... | 0.72774 |
| 40 | 5...N...(... | -0.78775 | 168 | c........... | 0.09423 | 296 | o...1....... | 0.72777 |
| 41 | ++++I---B2== | -0.77845 | 169 | c...c....... | 0.09687 | 297 | n...H...[... | 0.7463 |
| 42 | =...2....... | -0.77734 | 170 | C...@@...... | 0.1079 | 298 | C...#....... | 0.74953 |
| 43 | ++++N---S=== | -0.75661 | 171 | (...C...(... | 0.1093 | 299 | Cmax.2...... | 0.75992 |
| 44 | $10111000100 | -0.75023 | 172 | c...6....... | 0.11851 | 300 | n...n...1... | 0.7601 |
| 45 | N...C...3... | -0.74657 | 173 | c...4....... | 0.11857 | 301 | Nmax.2...... | 0.86278 |
| 46 | N........... | -0.73679 | 174 | c...2...N... | 0.11943 | 302 | Cl..(....... | 0.89864 |
| 47 | [...O...-... | -0.73563 | 175 | C6...AH.3... | 0.12308 | 303 | c...6...c... | 0.93456 |
| 48 | c...N....... | -0.73497 | 176 | 3...c...2... | 0.12708 | 304 | +...[...(... | 0.98309 |
| 49 | c...2...[... | -0.73423 | 177 | [...4...C... | 0.12728 | 305 | [...(...N... | 0.9896 |
| 50 | Cmax.5...... | -0.72426 | 178 | Cmax.4...... | 0.12827 | 306 | $10001000100 | 1.00847 |
| 51 | c...c...5... | -0.72406 | 179 | [........... | 0.1284 | 307 | Cmax.6...... | 1.04621 |
| 52 | ++++I---S=== | -0.72196 | 180 | C...C...4... | 0.12857 | 308 | c...C...(... | 1.04644 |
| 53 | 1...C...(... | -0.72123 | 181 | N...c...2... | 0.13039 | 309 | [...-...O... | 1.07123 |
| 54 | $10011100000 | -0.69141 | 182 | O...c...4... | 0.13295 | 310 | s...(...c... | 1.15717 |
| 55 | [...+...N... | -0.68374 | 183 | c...O...C... | 0.13694 | 311 | C...N...3... | 1.16009 |
| 56 | NOSP11000000 | -0.68038 | 184 | C5...AH.1... | 0.14041 | 312 | c...4...(... | 1.20578 |
| 57 | 2...C...1... | -0.66083 | 185 | BOND10100000 | 0.14631 | 313 | n...n....... | 1.21723 |
| 58 | c...c...6... | -0.6566 | 186 | C5...AH.2... | 0.15299 | 314 | $11111000000 | 1.21975 |
| 59 | c...4...N... | -0.65463 | 187 | O...C....... | 0.15813 | 315 | Nmax.0...... | 1.26596 |
| 60 | (...I...(... | -0.64795 | 188 | [...N....... | 0.16187 | 316 | [...3....... | 1.27299 |
| 61 | s...4....... | -0.6466 | 189 | N...(...C... | 0.16281 | 317 | [...c...(... | 1.27679 |
| 62 | S...(...=... | -0.64489 | 190 | C...C...C... | 0.16389 | 318 | S...(...O... | 1.28088 |
| 63 | 5...s...4... | -0.64298 | 191 | HALO00010000 | 0.16566 | 319 | Cl..c...1... | 1.30019 |
| 64 | c...I....... | -0.64262 | 192 | H...[...(... | 0.16583 | 320 | Smax.0...... | 1.32016 |
| 65 | O...C...3... | -0.63298 | 193 | c...c...3... | 0.17392 | 321 | c...5...c... | 1.34372 |
| 66 | 2...(....... | -0.63229 | 194 | C...C...2... | 0.17562 | 322 | 2...1....... | 1.3457 |
| 67 | C...c...6... | -0.6256 | 195 | C...5....... | 0.1766 | 323 | 4...c...3... | 1.34873 |
| 68 | C...=....... | -0.61835 | 196 | 6...(....... | 0.18382 | 324 | 2...c...1... | 1.36859 |
| 69 | c...4...O... | -0.61818 | 197 | C6...AH.2... | 0.18399 | 325 | C5....H.1... | 1.38644 |
| 70 | c...C....... | -0.61804 | 198 | O...=...2... | 0.1846 | 326 | c...3....... | 1.39699 |
| 71 | =...C...1... | -0.58084 | 199 | s...(....... | 0.18744 | 327 | (...C...#... | 1.44216 |
| 72 | n...O...(... | -0.57776 | 200 | c...4...C... | 0.19134 | 328 | C...4...=... | 1.4672 |
| 73 | =........... | -0.57708 | 201 | c...(...C... | 0.1917 | 329 | [...H...@@.. | 1.48219 |
| 74 | O...3...(... | -0.56738 | 202 | c...(...3... | 0.19206 | 330 | N...[...C... | 1.48438 |
| 75 | 3...c...(... | -0.55848 | 203 | O...=....... | 0.19378 | 331 | BOND11000000 | 1.5039 |
| 76 | 6........... | -0.54179 | 204 | @........... | 0.19386 | 332 | =...C...(... | 1.53035 |
| 77 | C...4...(... | -0.5384 | 205 | c...N...1... | 0.21365 | 333 | 4...s...3... | 1.535 |
| 78 | Omax.3...... | -0.50937 | 206 | -........... | 0.21596 | 334 | (...S...(... | 1.54457 |
| 79 | N...2...C... | -0.49101 | 207 | I...c...1... | 0.22322 | 335 | c...1...2... | 1.55673 |
| 80 | C...(...3... | -0.46121 | 208 | ++++B2--B3== | 0.22406 | 336 | S........... | 1.67988 |
| 81 | O...1....... | -0.45539 | 209 | =...O...(... | 0.22849 | 337 | Smax.1...... | 1.71959 |
| 82 | =...(....... | -0.45208 | 210 | C...[...(... | 0.23309 | 338 | H...[...4... | 1.78572 |
| 83 | ++++CL--O=== | -0.45193 | 211 | C...(...4... | 0.24041 | 339 | =...3....... | 1.88756 |
| 84 | HALO00000000 | -0.44933 | 212 | H...@...C... | 0.25141 | 340 | n...[....... | 1.99607 |
| 85 | n...4....... | -0.4367 | 213 | [...C...@... | 0.25142 | 341 | c...n...c... | 2.01477 |
| 86 | ++++S---B2== | -0.43364 | 214 | O...=...1... | 0.25361 | 342 | +........... | 2.03864 |
| 87 | c...(...O... | -0.43269 | 215 | BOND11100000 | 0.26488 | 343 | [...-....... | 2.0411 |
| 88 | O...C...C... | -0.43109 | 216 | c...C...C... | 0.26685 | 344 | [...+....... | 2.07352 |
| 89 | n...4...c... | -0.42923 | 217 | c...(...c... | 0.2671 | 345 | o...1...c... | 2.08335 |
| 90 | n...o...1... | -0.42496 | 218 | H...[...2... | 0.27159 | 346 | $10011000001 | 2.09945 |
| 91 | O...(...N... | -0.42317 | 219 | O...(...C... | 0.27463 | 347 | 2...3...(... | 2.10041 |
| 92 | 4...O...3... | -0.41687 | 220 | BOND10000000 | 0.29275 | 348 | C...3...=... | 2.12835 |
| 93 | C...2...(... | -0.40247 | 221 | [...(...C... | 0.29384 | 349 | [...4....... | 2.13723 |
| 94 | 3...s...(... | -0.40139 | 222 | H...[...3... | 0.29503 | 350 | ++++O---B3== | 2.14278 |
| 95 | n...n...c... | -0.39709 | 223 | [...2...C... | 0.29615 | 351 | N...4...C... | 2.1493 |
| 96 | 4........... | -0.39355 | 224 | (...N...(... | 0.29677 | 352 | 3...2....... | 2.16745 |
| 97 | C...C...(... | -0.36068 | 225 | (...Cl..(... | 0.29729 | 353 | C...(...1... | 2.1823 |
| 98 | NOSP11100000 | -0.35837 | 226 | C...1...(... | 0.29898 | 354 | NOSP01000000 | 2.2016 |
| 99 | c...3...[... | -0.35769 | 227 | [...H....... | 0.30229 | 355 | N...+....... | 2.22065 |
| 100 | ++++I---N=== | -0.34701 | 228 | 1...N...(... | 0.31374 | 356 | [...(...=... | 2.22348 |
| 101 | [...[...-... | -0.34392 | 229 | N...4....... | 0.31469 | 357 | 3........... | 2.24977 |
| 102 | c...1...C... | -0.32827 | 230 | C...4....... | 0.31534 | 358 | 2...N...(... | 2.26267 |
| 103 | 4...C...1... | -0.32115 | 231 | H...@@...... | 0.3164 | 359 | O...C...(... | 2.26559 |
| 104 | Omax.4...... | -0.29321 | 232 | c...[....... | 0.32096 | 360 | [...C...(... | 2.27056 |
| 105 | s...5....... | -0.25266 | 233 | 11011000000 | 0.32333 | 361 | n...c...1... | 2.27185 |
| 106 | c...1....... | -0.25182 | 234 | s...3...(... | 0.32375 | 362 | n...H....... | 2.29155 |
| 107 | [...[...N... | -0.24007 | 235 | Omax.2...... | 0.32424 | 363 | H...@....... | 2.29158 |
| 108 | 5........... | -0.23465 | 236 | o...n...c... | 0.32517 | 364 | C...(...6... | 2.33729 |
| 109 | C...@....... | -0.22299 | 237 | @@.......... | 0.33076 | 365 | [...C...C... | 2.33742 |
| 110 | c...2...c... | -0.20537 | 238 | C...C....... | 0.3313 | 366 | [...(....... | 2.33814 |
| 111 | s...3....... | -0.1849 | 239 | I........... | 0.33468 | 367 | O...-....... | 2.36367 |
| 112 | n...c....... | -0.16645 | 240 | [...1....... | 0.33646 | 368 | n...c...2... | 2.39016 |
| 113 | Cl.......... | -0.16602 | 241 | c...4...[... | 0.35354 | 369 | C...c...4... | 2.40302 |
| 114 | O...3....... | -0.15439 | 242 | O...=...C... | 0.36157 | 370 | $10001000000 | 2.44553 |
| 115 | C6...A..1... | -0.1511 | 243 | c...(...Cl.. | 0.36279 | 371 | [...1...(... | 2.45517 |
| 116 | #........... | -0.14538 | 244 | O...=...(... | 0.36748 | 372 | c...2...3... | 2.48287 |
| 117 | N...#...C... | -0.13699 | 245 | 2...c...(... | 0.36992 | 373 | [...N...(... | 2.52468 |
| 118 | C...(...2... | -0.10515 | 246 | ++++Cl--B2== | 0.37017 | 374 | Nmax.1...... | 2.52896 |
| 119 | o........... | -0.09694 | 247 | C...2....... | 0.37371 | 375 | N...3....... | 2.57166 |
| 120 | [...C....... | -0.09323 | 248 | 1...(....... | 0.37623 | 376 | 1...2...(... | 2.57298 |
| 121 | N...C...C... | -0.08583 | 249 | Nmax.4...... | 0.3822 | 377 | C...c...3... | 2.78119 |
| 122 | C........... | -0.08188 | 250 | C...N...2... | 0.385 | 378 | C6...A..3... | 3.10805 |
| 123 | c...3...C... | -0.06843 | 251 | c...4...c... | 0.38522 | 379 | ++++N---O=== | 4.18446 |
| 124 | s...4...c... | -0.06634 | 252 | C...O...1... | 0.38893 | 380 | ++++N---B2== | 4.32553 |
| 125 | C5......0... | -0.05339 | 253 | 3...(....... | 0.38929 | 381 | 2........... | 6.26212 |
| 126 | C...1...=... | -0.05167 | 254 | C...(...C... | 0.39034 | 382 | C...1...C... | 7.07316 |
| 127 | [...N...+... | -0.04516 | 255 | c...[...H... | 0.39132 | 383 | 1........... | 8.7943 |
| 128 | H...@@..C... | -0.04186 | 256 | [...[....... | 0.39408 |  |  |  |

**Table S2.** The results of Y-randomization test for all splits constructed based on TF1

|  | Split 1 | | |  | Split 2 | | |  | Split 3 | | |  | Split 4 | | |
| --- | --- | --- | --- | --- | --- | --- | --- | --- | --- | --- | --- | --- | --- | --- | --- |
| Run # | ATRN | PTRN | CAL |  | ATRN | PTRN | CAL |  | ATRN | PTRN | CAL |  | ATRN | PTRN | CAL |
| 1 | 0.007 | 0.021 | 0.119 |  | 0.061 | 0.000 | 0.000 |  | 0.024 | 0.002 | 0.137 |  | 0.028 | 0.002 | 0.001 |
| 2 | 0.017 | 0.002 | 0.093 |  | 0.008 | 0.007 | 0.033 |  | 0.003 | 0.176 | 0.179 |  | 0.043 | 0.135 | 0.042 |
| 3 | 0.001 | 0.000 | 0.008 |  | 0.004 | 0.010 | 0.040 |  | 0.030 | 0.014 | 0.007 |  | 0.005 | 0.000 | 0.236 |
| 4 | 0.049 | 0.234 | 0.000 |  | 0.135 | 0.130 | 0.004 |  | 0.002 | 0.007 | 0.007 |  | 0.097 | 0.011 | 0.008 |
| 5 | 0.013 | 0.001 | 0.000 |  | 0.001 | 0.006 | 0.004 |  | 0.016 | 0.157 | 0.032 |  | 0.026 | 0.006 | 0.004 |
| 6 | 0.007 | 0.143 | 0.004 |  | 0.018 | 0.032 | 0.001 |  | 0.149 | 0.032 | 0.047 |  | 0.022 | 0.025 | 0.080 |
| 7 | 0.000 | 0.028 | 0.003 |  | 0.041 | 0.023 | 0.056 |  | 0.046 | 0.124 | 0.021 |  | 0.020 | 0.101 | 0.028 |
| 8 | 0.006 | 0.005 | 0.030 |  | 0.077 | 0.008 | 0.015 |  | 0.016 | 0.009 | 0.017 |  | 0.027 | 0.012 | 0.144 |
| 9 | 0.045 | 0.139 | 0.030 |  | 0.016 | 0.016 | 0.004 |  | 0.006 | 0.080 | 0.168 |  | 0.005 | 0.004 | 0.041 |
| 10 | 0.057 | 0.011 | 0.080 |  | 0.021 | 0.096 | 0.002 |  | 0.008 | 0.001 | 0.270 |  | 0.057 | 0.011 | 0.025 |
| Average R^2^ | 0.020 | 0.058 | 0.037 |  | 0.038 | 0.033 | 0.016 |  | 0.030 | 0.060 | 0.088 |  | 0.033 | 0.031 | 0.061 |

**Table S3.** SMILES notations of isatin and indole derivatives, the compound set, their experimental, predicted pIC_50_, and applicability domain in four splits using TF1.

| No. | SMILES | pIC50 | Set | | | | DCW(T,N) | | | | Predicted pIC50 | | | | Applicability Domain | | | |
| --- | --- | --- | --- | --- | --- | --- | --- | --- | --- | --- | --- | --- | --- | --- | --- | --- | --- | --- |
|  |  |  | 1 | 2 | 3 | 4 | 1 | 2 | 3 | 4 | 1 | 2 | 3 | 4 | 1 | 2 | 3 | 4 |
| 1 | CCCCN1C(=O)C(=O)c2cc(I)ccc12 | 4.18 | + | - | - | - | 33.45 | 46.07 | 36.6 | 42.87 | 4.39 | 4.4 | 4.32 | 4.63 | N | Y | Y | Y |
| 2 | Ic1ccc2N(Cc3ccc4ccccc4c3)C(=O)C(=O)c2c1 | 5.96 | + | + | + | + | 55.57 | 62.64 | 46.44 | 52.53 | 5.66 | 6.1 | 5.53 | 5.6 | Y | Y | Y | Y |
| 3 | CN1C(=O)C(=O)c2cc(ccc12)C(=O)N | 4.15 | + | + | * | + | 31 | 45.4 | 36.40 | 39.97 | 4.25 | 4.33 | 4.29 | 4.33 | Y | Y | Y | Y |
| 4 | CCCN1C(=O)C(=O)c2cc(ccc12)C(=O)N | 4.60 | - | # | + | - | 31.58 | 48.09 | 37.93 | 42.90 | 4.29 | 4.6 | 4.48 | 4.63 | Y | Y | Y | Y |
| 5 | CCCCN1C(=O)C(=O)c2cc(ccc12)C(=O)N | 4.72 | * | * | * | * | 31.99 | 49.63 | 39.65 | 45.44 | 4.31 | 4.75 | 4.69 | 4.88 | Y | Y | Y | Y |
| 6 | NC(=O)c1ccc2N(Cc3ccccc3)C(=O)C(=O)c2c1 | 4.90 | # | + | + | + | 43.38 | 52.32 | 40.64 | 44.92 | 4.96 | 5.01 | 4.81 | 4.83 | Y | Y | Y | Y |
| 7 | Cc1onc(C)c1CN2C(=O)C(=O)c3cc(ccc23)C#N | 5.14 | + | * | # | * | 49.08 | 63.95 | 39.97 | 45.13 | 5.29 | 6.14 | 4.73 | 4.85 | N | N | Y | Y |
| 8 | Fc1ccc(CN2C(=O)C(=O)c3cc(I)ccc23)c(Cl)c1 | 5.03 | - | + | * | # | 48 | 52.06 | 38.09 | 45.09 | 5.23 | 4.98 | 4.50 | 4.85 | N | N | Y | Y |
| 9 | Ic1ccc2N(CC3COc4ccccc4O3)C(=O)C(=O)c2c1 | 4.87 | + | + | # | # | 40.03 | 51.59 | 43.97 | 48.26 | 4.77 | 4.94 | 5.22 | 5.17 | N | N | Y | Y |
| 10 | O=C1N(Cc2cc3ccccc3s2)c4ccccc4C1=O | 4.90 | * | * | * | # | 34.38 | 45.44 | 37.73 | 39.08 | 4.45 | 4.34 | 4.46 | 4.25 | Y | Y | Y | Y |
| 11 | [O-][N+](=O)c1cccc2C(=O)C(=O)N(Cc3cc4ccccc4s3)c12 | 5.7 | + | * | # | + | 62.93 | 57.75 | 47.81 | 56.24 | 6.08 | 5.54 | 5.69 | 5.97 | N | Y | Y | N |
| 12 | Brc1cccc2C(=O)C(=O)N(Cc3cc4ccccc4s3)c12 | 6 | - | + | + | # | 51.14 | 64.74 | 48.11 | 49.14 | 5.41 | 6.21 | 5.73 | 5.25 | Y | N | Y | Y |
| 13 | Fc1ccc2N(Cc3cc4ccccc4s3)C(=O)C(=O)c2c1 | 5.31 | - | - | * | * | 44.49 | 55.92 | 36.14 | 44.64 | 5.03 | 5.36 | 4.26 | 4.80 | Y | Y | Y | Y |
| 14 | Clc1cccc2N(Cc3cc4ccccc4s3)C(=O)C(=O)c12 | 4.95 | # | # | - | + | 45.78 | 51.82 | 44.01 | 43.65 | 5.1 | 4.96 | 5.23 | 4.70 | Y | Y | Y | Y |
| 15 | Ic1ccc2N(C\C=C\c3cc4ccccc4s3)C(=O)C(=O)c2c1 | 4.63 | - | - | + | + | 39.68 | 53.02 | 37.92 | 44.49 | 4.75 | 5.07 | 4.48 | 4.79 | Y | Y | N | N |
| 16 | Clc1ccc(NC(=O)c2ccc(CN3C(=O)C(=O)c4cc(I)ccc34)s2)cc1 | 4.90 | * | * | * | # | 35.42 | 57.48 | 34.55 | 42.5 | 4.51 | 5.51 | 4.07 | 4.59 | N | N | Y | Y |
| 17 | Ic1ccc2N(Cc3ccc(s3)C(=O)N4CCCCC4)C(=O)C(=O)c2c1 | 4.76 | + | # | - | - | 43.66 | 53.50 | 40.09 | 46.37 | 4.98 | 5.12 | 4.75 | 4.98 | N | Y | Y | Y |
| 18 | CN1CCN(CC1)S(=O)(=O)c2ccc3NC(=O)C(=O)c3c2 | 4.12 | # | - | # | - | 39.37 | 49.63 | 33.82 | 42.59 | 4.73 | 4.75 | 3.98 | 4.6 | Y | Y | Y | Y |
| 19 | Clc1cccc(CN2CCN(CC2)S(=O)(=O)c3ccc4NC(=O)C(=O)c4c3)c1 | 4.5 | - | # | * | * | 36.44 | 52.07 | 36.63 | 37.89 | 4.56 | 4.98 | 4.32 | 4.13 | N | Y | N | N |
| 20 | COc1cc(CN2CCN(CC2)S(=O)(=O)c3ccc4NC(=O)C(=O)c4c3)cc(OC)c1OC | 4.49 | + | * | + | + | 36.63 | 57.92 | 36.66 | 42.22 | 4.58 | 5.55 | 4.33 | 4.56 | N | Y | N | N |
| 21 | O=C1Nc2ccc(cc2C1=O)S(=O)(=O)N3CCN(CCc4ccccc4)CC3 | 4.46 | + | + | + | + | 41.50 | 49.95 | 39.09 | 40.46 | 4.85 | 4.78 | 4.62 | 4.38 | Y | N | Y | N |
| 22 | O=C(N1CCN(CC1)S(=O)(=O)c2ccc3NC(=O)C(=O)c3c2)c4occc4 | 5 | + | * | * | - | 41.78 | 54.88 | 35.07 | 48.51 | 4.87 | 5.25 | 4.13 | 5.19 | Y | Y | Y | Y |
| 23 | O=C1Nc2ccc(cc2C1=O)S(=O)(=O)N3CCN(CC3)c4ccccn4 | 4.29 | + | - | + | - | 35.33 | 50.26 | 36.36 | 43.38 | 4.50 | 4.81 | 4.29 | 4.68 | Y | N | Y | N |
| 24 | O=C1Nc2ccc(cc2C1=O)S(=O)(=O)N3CCCCC3 | 5.35 | + | + | + | + | 45.32 | 55.56 | 43.75 | 49.21 | 5.07 | 5.32 | 5.2 | 5.26 | Y | N | Y | N |
| 25 | O=C1Nc2ccc(cc2C1=O)S(=O)(=O)N3CCOCC3 | 4.9 | * | * | + | - | 44.89 | 54.87 | 40.51 | 45.1 | 5.05 | 5.25 | 4.8 | 4.85 | Y | N | Y | N |
| 26 | CC1CCN(CC1)S(=O)(=O)c2ccc3NC(=O)C(=O)c3c2 | 5.93 | * | + | # | - | 56.74 | 59.02 | 43.71 | 56.5 | 5.73 | 5.66 | 5.19 | 5.99 | Y | Y | Y | Y |
| 27 | CC1CCCCN1S(=O)(=O)c2ccc3NC(=O)C(=O)c3c2 | 5.65 | * | - | # | + | 50.50 | 57.85 | 42.59 | 53.01 | 5.37 | 5.54 | 5.05 | 5.64 | Y | Y | Y | Y |
| 28 | CC1CC(C)CN(C1)S(=O)(=O)c2ccc3NC(=O)C(=O)c3c2 | 5.37 | + | * | - | * | 52.80 | 53.42 | 43.94 | 50.08 | 5.50 | 5.11 | 5.22 | 5.35 | Y | Y | Y | Y |
| 29 | CN1CCN(CC1)S(=O)(=O)c2ccc3N(C)C(=O)C(=O)c3c2 | 4.93 | + | * | * | - | 35.42 | 47.37 | 34.45 | 42.72 | 4.51 | 4.53 | 4.06 | 4.61 | Y | Y | Y | Y |
| 30 | CN1CCN(CC1)S(=O)(=O)c2ccc3N(Cc4ccccc4)C(=O)C(=O)c3c2 | 4.17 | - | + | + | # | 38.03 | 48.01 | 38.01 | 42.28 | 4.66 | 4.59 | 4.49 | 4.57 | Y | Y | Y | Y |
| 31 | CN1CCN(CC1)S(=O)(=O)c2ccc3N(Cc4ccc5ccccc5c4)C(=O)C(=O)c3c2 | 4.08 | - | - | - | - | 32.81 | 48.36 | 34.24 | 42 | 4.36 | 4.62 | 4.03 | 4.54 | Y | Y | Y | Y |
| 32 | O=C1N(Cc2ccc3ccccc3c2)c4ccc(cc4C1=O)S(=O)(=O)N5CCN(CCc6ccccc6)CC5 | 4.86 | + | # | * | - | 37.74 | 51.16 | 36.34 | 45.06 | 4.64 | 4.89 | 4.29 | 4.85 | N | Y | Y | Y |
| 33 | O=C1N(Cc2ccc3ccccc3c2)c4ccc(cc4C1=O)S(=O)(=O)N5CCN(CC5)c6ccccn6 | 5.26 | * | - | # | * | 37.98 | 55.06 | 35.7 | 47.30 | 4.65 | 5.27 | 4.21 | 5.07 | Y | Y | Y | Y |
| 34 | CN1C(=O)C(=O)c2cc(ccc12)S(=O)(=O)N3CCOCC3 | 5 | * | - | # | + | 41 | 54.22 | 40.57 | 46.06 | 4.83 | 5.19 | 4.81 | 4.95 | Y | Y | Y | Y |
| 35 | O=C1N(Cc2ccccc2)c3ccc(cc3C1=O)S(=O)(=O)N4CCOCC4 | 4.86 | + | + | + | * | 45.68 | 51.66 | 40.60 | 45 | 5.09 | 4.94 | 4.81 | 4.84 | Y | Y | Y | Y |
| 36 | O=C1N(Cc2ccc3ccccc3c2)c4ccc(cc4C1=O)S(=O)(=O)N5CCOCC5 | 4.4 | # | - | + | - | 41.64 | 53.33 | 39.57 | 46.01 | 4.86 | 5.10 | 4.68 | 4.94 | Y | Y | Y | Y |
| 37 | CC1CCN(CC1)S(=O)(=O)c2ccc3N(Cc4ccccc4)C(=O)C(=O)c3c2 | 5.98 | + | + | * | * | 55.40 | 57.4 | 47.90 | 56.18 | 5.65 | 5.50 | 5.70 | 5.96 | Y | Y | Y | Y |
| 38 | CC1CCN(CC1)S(=O)(=O)c2ccc3N(Cc4ccc5ccccc5c4)C(=O)C(=O)c3c2 | 5.77 | * | + | - | + | 50.18 | 57.75 | 44.13 | 55.91 | 5.35 | 5.53 | 5.24 | 5.94 | Y | Y | Y | Y |
| 39 | CC1CCN(CC1)S(=O)(=O)c2ccc3N(C)C(=O)C(=O)c3c2 | 4.75 | * | - | - | * | 52.79 | 56.76 | 44.34 | 56.63 | 5.5 | 5.44 | 5.27 | 6.01 | Y | Y | Y | Y |
| 40 | CC1CC(C)CN(C1)S(=O)(=O)c2ccc3N(Cc4ccccc4)C(=O)C(=O)c3c2 | 5.55 | * | # | + | * | 51.46 | 51.79 | 48.13 | 49.76 | 5.42 | 4.96 | 5.73 | 5.32 | Y | Y | Y | Y |
| 41 | CC1CC(C)CN(C1)S(=O)(=O)c2ccc3N(Cc4ccc5ccccc5c4)C(=O)C(=O)c3c2 | 5.33 | # | + | - | - | 46.24 | 52.14 | 44.36 | 49.49 | 5.12 | 4.99 | 5.27 | 5.29 | Y | Y | Y | Y |
| 42 | COc1cccc2[nH]c(cc12)C(=O)N[C@@H](CC(C)C)C(=O)N[C@@H](C[C@@H]3CCNC3=O)C(=O)COC(=O)C | 6.66 | # | # | - | + | 77.50 | 71.94 | 59.34 | 62.95 | 6.91 | 6.91 | 7.11 | 6.64 | Y | Y | Y | Y |
| 43 | COc1cccc2[nH]c(cc12)C(=O)N[C@@H](CC(C)C)C(=O)N[C@@H](C[C@@H]3CCNC3=O)C(=O)COC(=O)C4CC4 | 6.74 | * | # | # | # | 78.16 | 67.78 | 56.85 | 63.35 | 6.95 | 6.51 | 6.80 | 6.68 | Y | Y | Y | Y |
| 44 | COc1cccc2[nH]c(cc12)C(=O)N[C@@H](CC(C)C)C(=O)N[C@@H](C[C@@H]3CCNC3=O)C(=O)COC(=O)C(C)(C)C | 6.64 | * | + | # | + | 72.93 | 72.13 | 60.01 | 65.46 | 6.65 | 6.93 | 7.19 | 6.89 | Y | Y | Y | Y |
| 45 | COc1cccc2[nH]c(cc12)C(=O)N[C@@H](CC(C)C)C(=O)N[C@@H](C[C@@H]3CCNC3=O)C(=O)COC(=O)c4ccccc4 | 7.07 | * | - | * | * | 75.57 | 72.89 | 61.79 | 67.49 | 6.80 | 7.01 | 7.41 | 7.1 | Y | Y | Y | Y |
| 46 | COc1ccc(cc1)C(=O)OCC(=O)[C@H](C[C@@H]2CCNC2=O)NC(=O)[C@H](CC(C)C)NC(=O)c3cc4c(OC)cccc4[nH]3 | 7.10 | - | * | - | # | 76.20 | 75.59 | 57.08 | 65.09 | 6.84 | 7.27 | 6.83 | 6.86 | Y | Y | N | Y |
| 47 | COc1cccc2[nH]c(cc12)C(=O)N[C@@H](CC(C)C)C(=O)N[C@@H](C[C@@H]3CCNC3=O)C(=O)COC(=O)c4ccc(C)cc4 | 7.06 | * | - | # | + | 76.93 | 74.89 | 60.07 | 65.54 | 6.88 | 7.20 | 7.2 | 6.90 | Y | Y | Y | Y |
| 48 | COc1cccc2[nH]c(cc12)C(=O)N[C@@H](CC(C)C)C(=O)N[C@@H](C[C@@H]3CCNC3=O)C(=O)COC(=O)c4ccc(cc4)C#N~ | 7.28 | + | - | - | # | 84.47 | 82.99 | 58.3 | 69.49 | 7.31 | 7.99 | 6.98 | 7.3 | N | N | Y | Y |
| 49 | COc1cccc2[nH]c(cc12)C(=O)N[C@@H](CC(C)C)C(=O)N[C@@H](C[C@@H]3CCNC3=O)C(=O)COC(=O)c4ccc(F)cc4 | 7.09 | * | # | * | # | 78.38 | 76.94 | 58.34 | 67.29 | 6.96 | 7.40 | 6.98 | 7.08 | Y | Y | Y | Y |
| 50 | COc1cccc2[nH]c(cc12)C(=O)N[C@@H](CC(C)C)C(=O)N[C@@H](C[C@@H]3CCNC3=O)C(=O)COC(=O)c4ccc(Cl)cc4 | 7.01 | + | * | # | - | 80.68 | 71.68 | 63.79 | 70.3 | 7.09 | 6.89 | 7.65 | 7.38 | Y | Y | Y | Y |
| 51 | COc1cccc2[nH]c(cc12)C(=O)N[C@@H](CC(C)C)C(=O)N[C@@H](C[C@@H]3CCNC3=O)C(=O)COC(=O)c4c(C)cccc4C | 7.13 | # | * | + | * | 74.46 | 70.43 | 59.28 | 65.07 | 6.74 | 6.77 | 7.1 | 6.85 | Y | Y | Y | Y |
| 52 | COc1cccc(OC)c1C(=O)OCC(=O)[C@H](C[C@@H]2CCNC2=O)NC(=O)[C@H](CC(C)C)NC(=O)c3cc4c(OC)cccc4[nH]3 | 6.69 | + | - | * | - | 72.96 | 74.4 | 62.83 | 63.35 | 6.65 | 7.15 | 7.53 | 6.68 | N | Y | N | Y |
| 53 | COc1cccc2[nH]c(cc12)C(=O)N[C@@H](CC(C)C)C(=O)N[C@@H](C[C@@H]3CCNC3=O)C(=O)COC(=O)c4ccccc4C#N | 7.77 | - | + | - | - | 80.83 | 80.15 | 58.84 | 70.38 | 7.10 | 7.71 | 7.04 | 7.39 | Y | N | Y | Y |
| 54 | COCC(=O)[C@H](C[C@@H]1CCNC1=O)NC(=O)[C@H](CC(C)(C)C)NC(=O)c2cc3c(OC)cccc3[nH]2 | 7.46 | # | * | * | # | 73.24 | 78.81 | 59.31 | 76.35 | 6.67 | 7.58 | 7.10 | 7.99 | Y | Y | Y | Y |
| 55 | COc1cccc2[nH]c(cc12)C(=O)N(C)[C@@H](CC(C)C)C(=O)N[C@@H](C[C@@H]3CCNC3=O)C(=O)CO | 7.08 | * | * | + | * | 75.71 | 76.37 | 58.79 | 66.23 | 6.81 | 7.35 | 7.04 | 6.97 | Y | Y | Y | Y |
| 56 | CCCC[C@H](NC(=O)c1cc2c(OC)cccc2[nH]1)C(=O)N[C@@H](C[C@@H]3CCNC3=O)C(=O)CO | 7.7 | + | + | # | + | 87.06 | 82.17 | 63.93 | 75.16 | 7.46 | 7.91 | 7.67 | 7.87 | Y | Y | Y | Y |
| 57 | COc1cccc2[nH]c(cc12)C(=O)N[C@@H](CCC(C)C)C(=O)N[C@@H](C[C@@H]3CCNC3=O)C(=O)CO | 7.47 | # | # | + | # | 78.05 | 75.34 | 59.59 | 67.08 | 6.94 | 7.25 | 7.14 | 7.06 | Y | Y | Y | Y |
| 58 | COc1cccc2[nH]c(cc12)C(=O)N[C@@H](CC3CCCCC3)C(=O)N[C@@H](C[C@@H]4CCNC4=O)C(=O)CO | 7.36 | # | * | * | * | 77.91 | 78.39 | 61.82 | 76.67 | 6.94 | 7.54 | 7.41 | 8.02 | Y | N | Y | Y |
| 59 | COc1cccc2[nH]c(cc12)C(=O)N[C@@H](Cc3ccccc3)C(=O)N[C@@H](C[C@@H]4CCNC4=O)C(=O)CO | 6.99 | + | + | * | - | 83 | 74.50 | 59.86 | 68.53 | 7.23 | 7.16 | 7.17 | 7.20 | Y | N | Y | Y |
| 60 | CC(C)(C)C[C@H](NC(=O)c1cc2ccccc2[nH]1)C(=O)N[C@@H](C[C@@H]3CCNC3=O)C(=O)CO | 7.7 | - | + | - | + | 78.62 | 73.92 | 60.25 | 67.94 | 6.98 | 7.11 | 7.22 | 7.14 | Y | Y | Y | Y |
| 61 | COCC(=O)[C@H](C[C@@H]1CCNC1=O)NC(=O)[C@H](CC(C)(C)C)NC(=O)c2cc3ccccc3[nH]2 | 6.98 | # | # | * | * | 71.40 | 72.11 | 58.77 | 73.68 | 6.56 | 6.93 | 7.04 | 7.72 | Y | Y | Y | Y |
| 62 | CCOCC(=O)[C@H](C[C@@H]1CCNC1=O)NC(=O)[C@H](CC(C)(C)C)NC(=O)c2cc3ccccc3[nH]2 | 6.95 | # | # | * | * | 71.22 | 73.36 | 58.61 | 73.05 | 6.55 | 7.05 | 7.02 | 7.66 | Y | Y | Y | Y |
| 63 | COCC(=O)[C@H](C[C@@H]1CCNC1=O)NC(=O)[C@H](CC(C)C)NC(=O)c2cc3c(OC)cccc3[nH]2 | 7.28 | - | * | - | - | 74.95 | 75.96 | 58.56 | 69.63 | 6.77 | 7.31 | 7.01 | 7.31 | Y | Y | Y | Y |
| 64 | CC(C)C[C@H](NC(=O)c1cc2ccccc2[nH]1)C(=O)N[C@@H](C[C@@H]3CCNC3=O)C(=O)CO | 7.42 | # | * | # | * | 80.33 | 71.07 | 59.51 | 61.23 | 7.07 | 6.83 | 7.13 | 6.47 | Y | Y | Y | Y |
| 65 | COCC(=O)[C@H](C[C@@H]1CCNC1=O)NC(=O)[C@H](CC(C)C)NC(=O)c2cc3ccccc3[nH]2 | 6.88 | - | # | + | + | 73.11 | 69.26 | 58.03 | 66.96 | 6.66 | 6.65 | 6.95 | 7.05 | Y | Y | Y | Y |
| 66 | Clc1cncc(OC(=O)c2cc3ccccc3[nH]2)c1 | 7.19 | * | + | - | * | 69.81 | 76.39 | 56.02 | 67.11 | 6.47 | 7.35 | 6.7 | 7.06 | Y | Y | N | Y |
| 67 | O=C(On1nnc2ccccc12)c3ccc4[nH]ccc4c3 | 6.7 | + | - | + | # | 76.45 | 69.92 | 57.44 | 58.89 | 6.85 | 6.72 | 6.87 | 6.23 | N | Y | N | Y |
| 68 | Clc1cncc(OC(=O)c2ccc3[nH]ccc3c2)c1 | 6.51 | + | * | + | + | 70.50 | 74.19 | 55.35 | 64.15 | 6.51 | 7.13 | 6.62 | 6.76 | Y | Y | N | Y |
| 69 | CC(=O)n1ccc2cc(ccc12)C(=O)Oc3cncc(Cl)c3 | 6.4 | - | - | - | + | 62.12 | 68.87 | 51.42 | 60.81 | 6.03 | 6.62 | 6.14 | 6.43 | Y | Y | N | Y |
| 70 | Cc1ccc(cc1)S(=O)(=O)n2ccc3cc(ccc23)C(=O)Oc4cncc(Cl)c4 | 6.43 | - | * | - | - | 59.88 | 63.66 | 52.43 | 61.9 | 5.90 | 6.11 | 6.26 | 6.54 | Y | Y | N | Y |
| 71 | [O-][N+](=O)c1cccc(c1)S(=O)(=O)n2ccc3cc(ccc23)C(=O)Oc4cncc(Cl)c4 | 7.05 | - | # | + | * | 77.37 | 68.36 | 58.89 | 75.37 | 6.90 | 6.57 | 7.05 | 7.89 | N | Y | N | N |
| 72 | Clc1cncc(OC(=O)c2ccc3cc[nH]c3c2)c1 | 6.64 | - | + | * | + | 72.3 | 73.42 | 52.77 | 64.56 | 6.61 | 7.06 | 6.30 | 6.80 | Y | Y | Y | Y |
| 73 | Clc1cncc(OC(=O)c2cccc3[nH]ccc23)c1 | 7.52 | - | + | - | + | 76.02 | 75.99 | 60.15 | 67.94 | 6.83 | 7.31 | 7.21 | 7.14 | Y | Y | N | Y |
| 74 | CC(=O)n1ccc2c(cccc12)C(=O)Oc3cncc(Cl)c3 | 5.97 | # | # | + | # | 60.65 | 67.81 | 51.07 | 62.54 | 5.95 | 6.51 | 6.09 | 6.60 | Y | Y | N | Y |
| 75 | Clc1cncc(OC(=O)c2cccc3cc[nH]c23)c1 | 7.1 | * | + | * | # | 77.55 | 73.92 | 56.77 | 69.28 | 6.91 | 7.11 | 6.79 | 7.28 | Y | N | Y | Y |
| 76 | CC(C)C[C@H](NC(=O)c1cc2ccccc2[nH]1)C(=O)N[C@@H](C[C@@H]3CCNC3=O)C(=O)c4nc5ccccc5s4 | 5.82 | + | * | # | * | 55.74 | 60.14 | 47.60 | 52.30 | 5.67 | 5.77 | 5.67 | 5.57 | Y | Y | Y | Y |
| 77 | COc1ccc2[nH]c(cc2c1)C(=O)N[C@@H](CC(C)C)C(=O)N[C@@H](C[C@@H]3CCNC3=O)C(=O)c4nc5ccccc5s4 | 5.34 | * | - | # | + | 55.77 | 58.86 | 48.41 | 50.10 | 5.67 | 5.64 | 5.77 | 5.35 | Y | Y | Y | Y |
| 78 | CC(C)C[C@H](NC(=O)c1cc2cc(Cl)ccc2[nH]1)C(=O)N[C@@H](C[C@@H]3CCNC3=O)C(=O)c4nc5ccccc5s4 | 5.32 | # | - | + | - | 60.86 | 58.93 | 49.05 | 52.35 | 5.96 | 5.65 | 5.85 | 5.58 | Y | Y | Y | Y |
| 79 | COc1cccc2[nH]c(cc12)C(=O)N[C@@H](CC(C)C)C(=O)N[C@@H](C[C@@H]3CCNC3=O)C(=O)c4nc5ccccc5s4 | 6.13 | + | + | - | # | 54.28 | 59.42 | 45.92 | 54.31 | 5.58 | 5.7 | 5.47 | 5.77 | Y | Y | Y | Y |
| 80 | CC(C)COc1cccc2[nH]c(cc12)C(=O)N[C@@H](CC(C)C)C(=O)N[C@@H](C[C@@H]3CCNC3=O)C(=O)c4nc5ccccc5s4 | 5.28 | - | # | - | - | 53.46 | 57.48 | 46.08 | 50.94 | 5.54 | 5.51 | 5.48 | 5.44 | Y | Y | Y | Y |
| 81 | CC(C)C[C@H](NC(=O)c1cc2c(O)cccc2[nH]1)C(=O)N[C@@H](C[C@@H]3CCNC3=O)C(=O)c4nc5ccccc5s4 | 5.82 | - | * | * | + | 57.06 | 59.73 | 46.44 | 56.08 | 5.74 | 5.73 | 5.52 | 5.95 | Y | Y | Y | Y |

**Table S4.** The affinity of nine conformations docked into SARS-COV-1 3CLpro (PDB: 1UK4 and 6XHO) for compounds 12 and 53

|  | Affinity (kcal mol^-1^) | |
| --- | --- | --- |
| Pose | Compound 12 | Compound 53 |
| 1 | -8.1 | -9.7 |
| 2 | -7.5 | -9.4 |
| 3 | -7.2 | -9.0 |
| 4 | -7.0 | -9.0 |
| 5 | -6.7 | -9.0 |
| 6 | -6.7 | -9.0 |
| 7 | -6.6 | -8.7 |
| 8 | -6.6 | -8.7 |
| 9 | -6.6 | -8.7 |


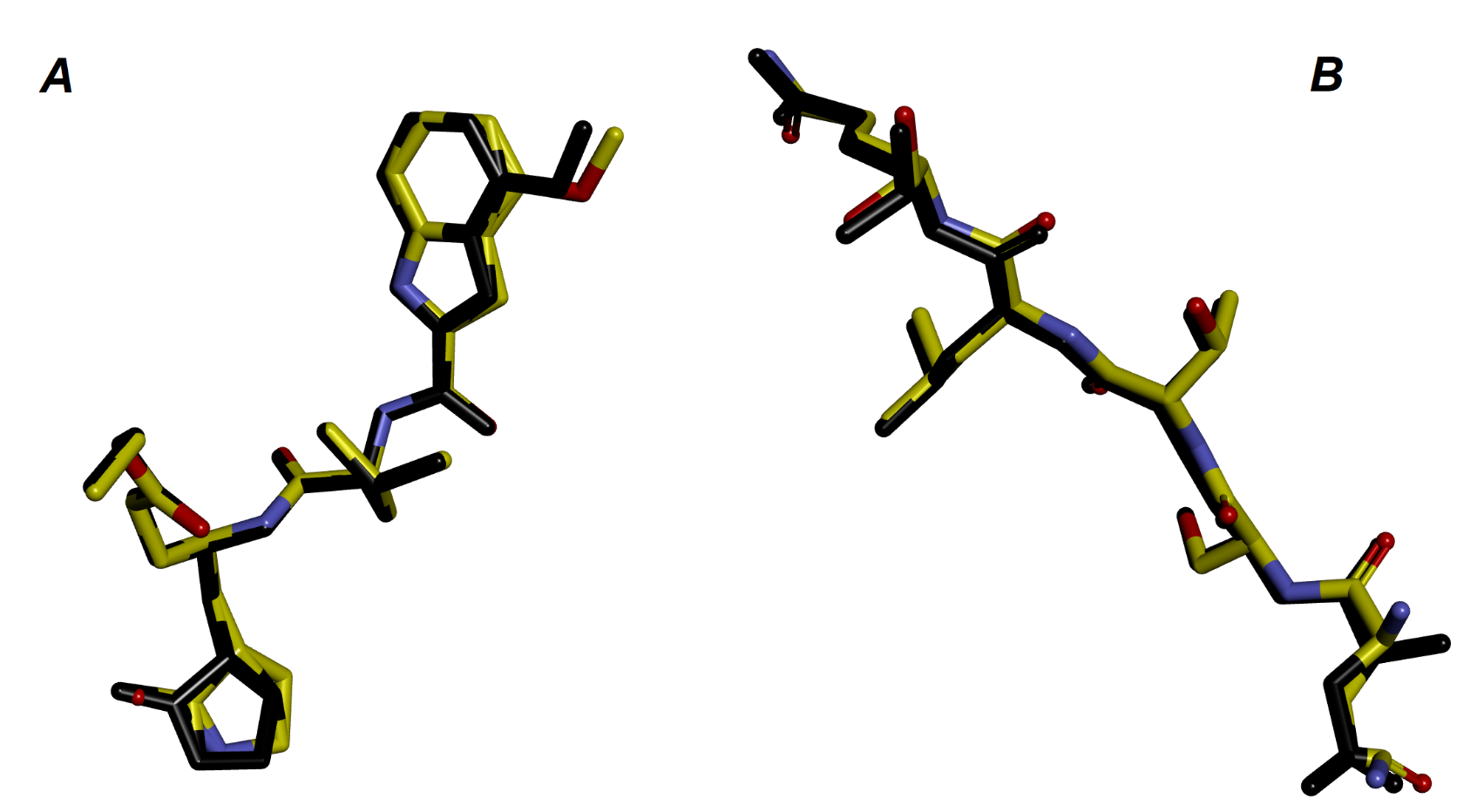


**Figure 1S**. 3D superposition of original (black) and re-docked (yellow) (A) V34 ligand in the 6XHO (RMSD=0.14 Å), (A) 5-mer peptide ligand in the 1UK4 (RMSD=1.1 Å)


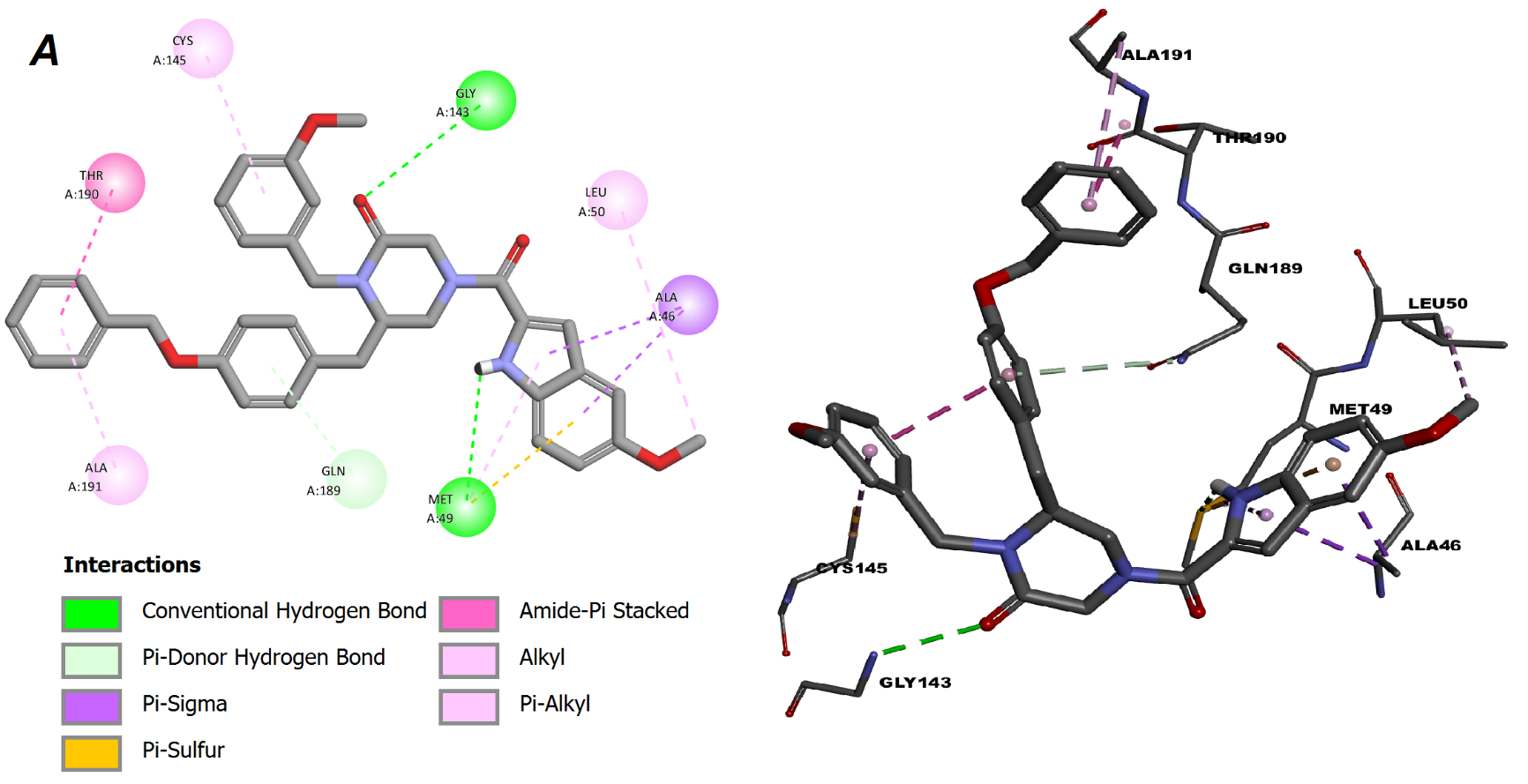


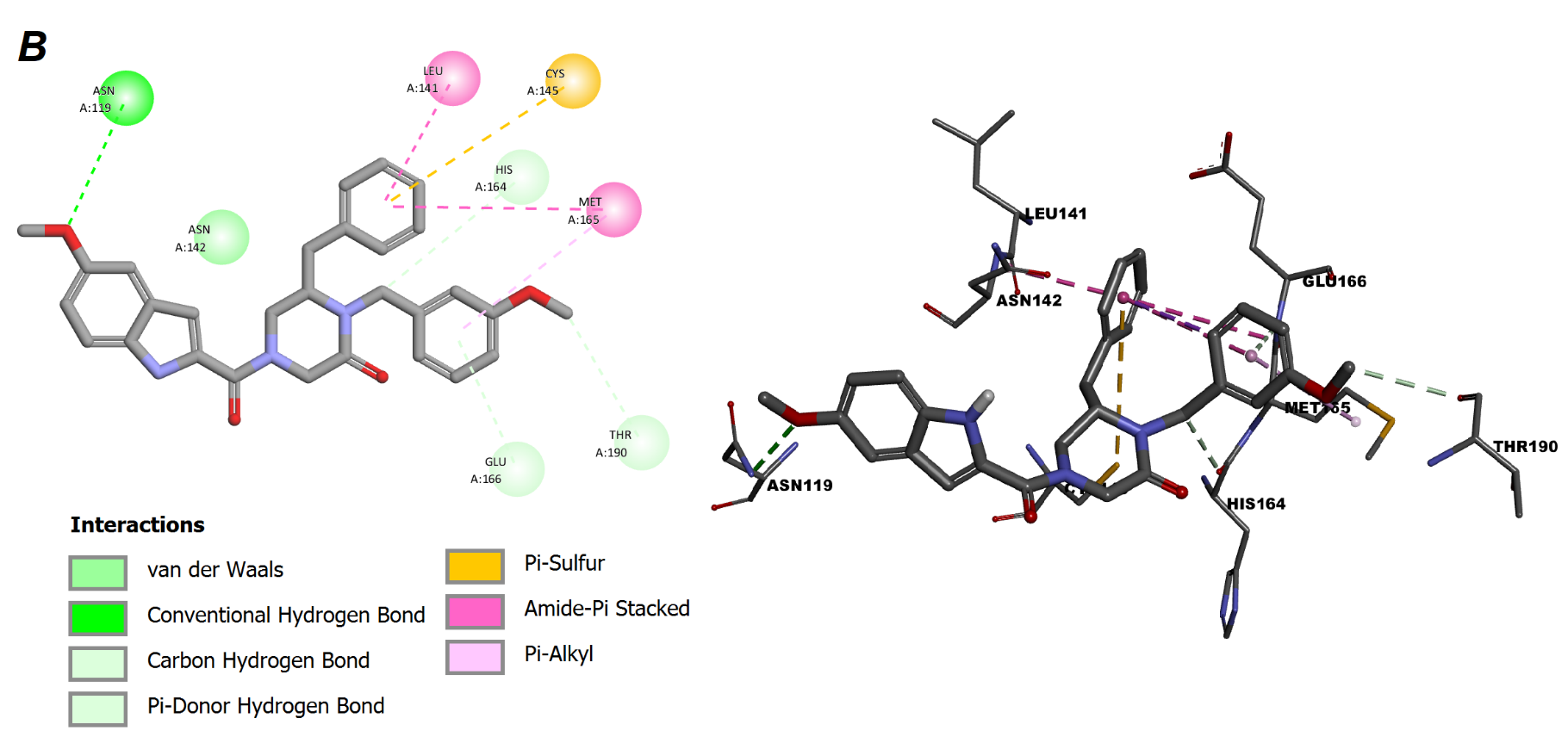


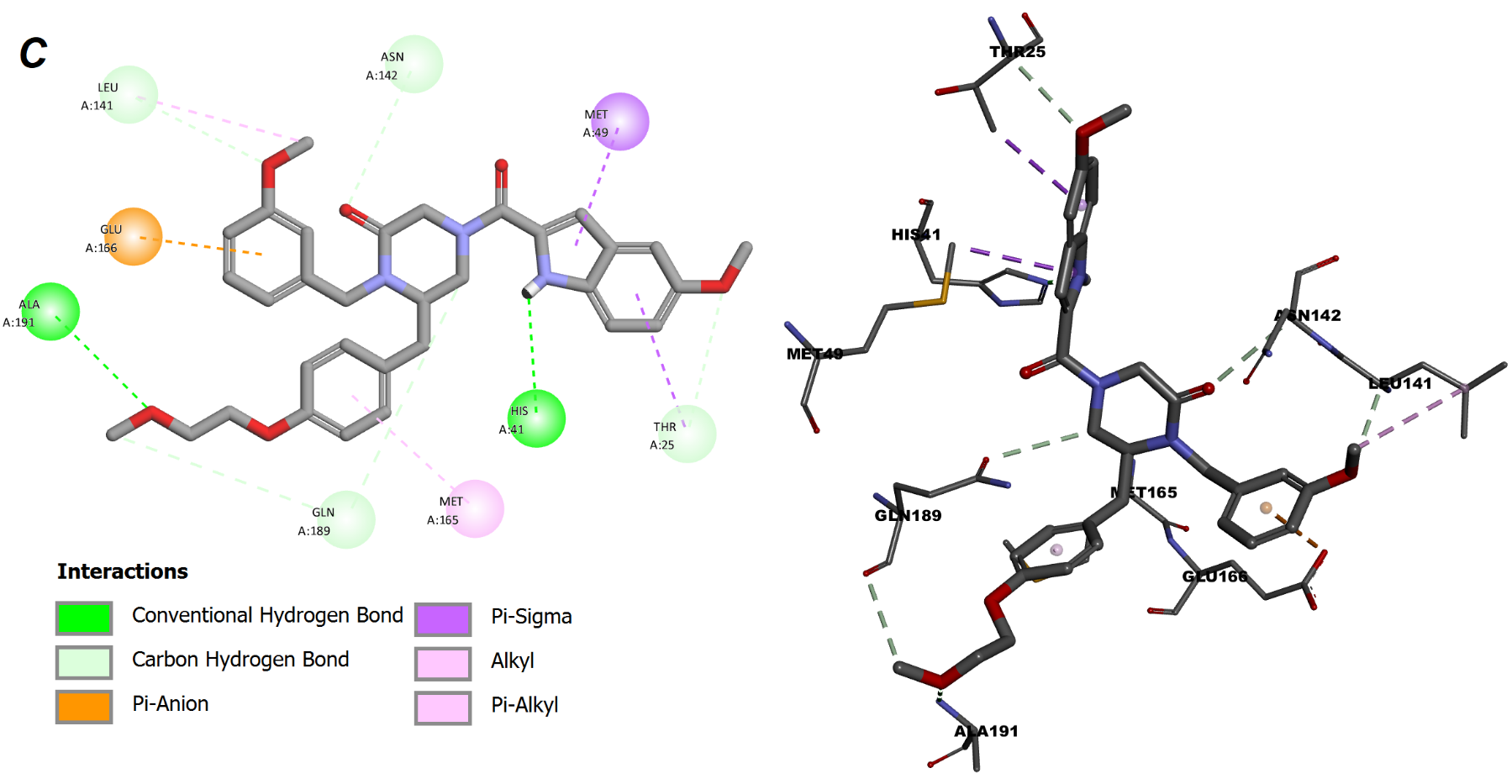


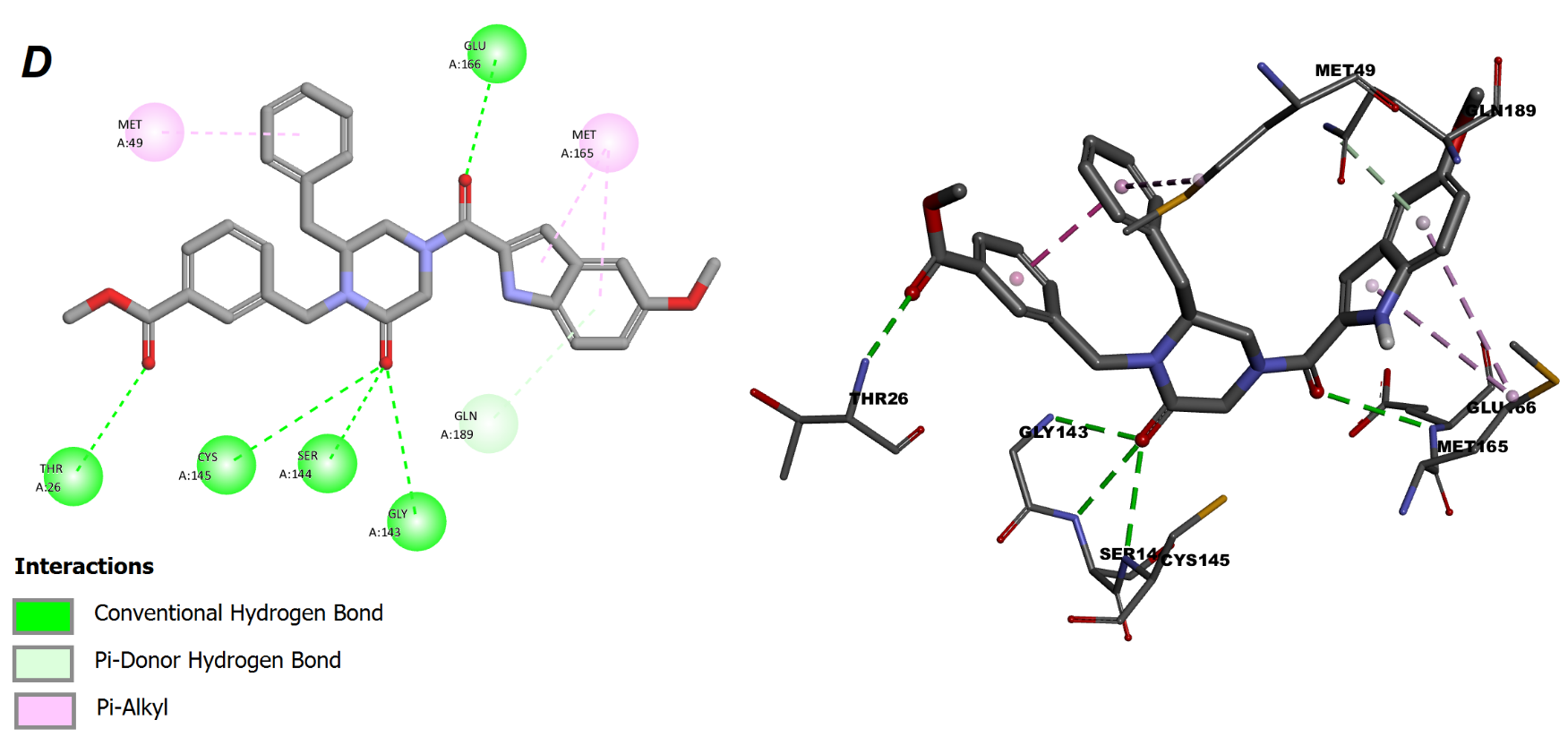


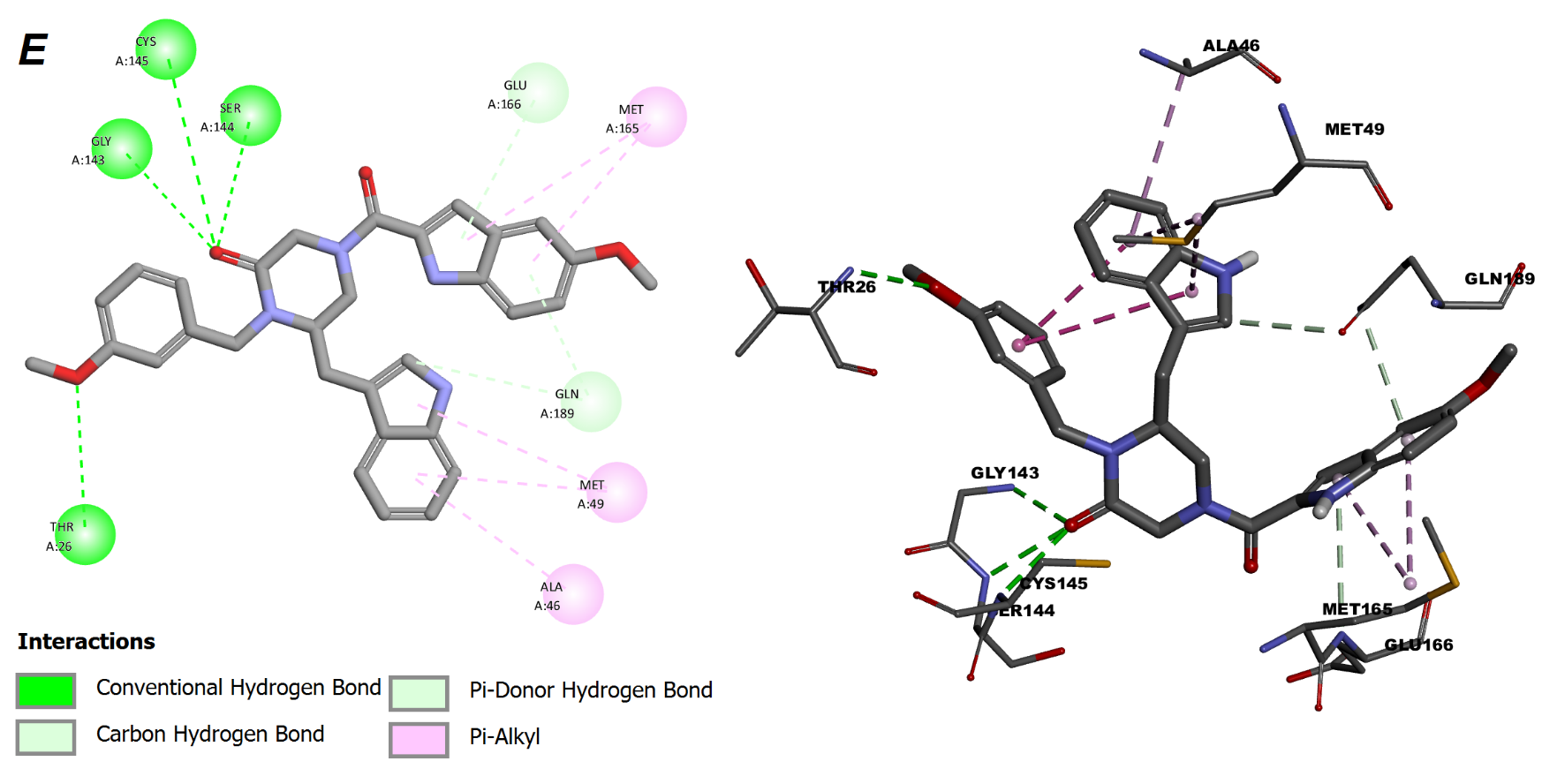


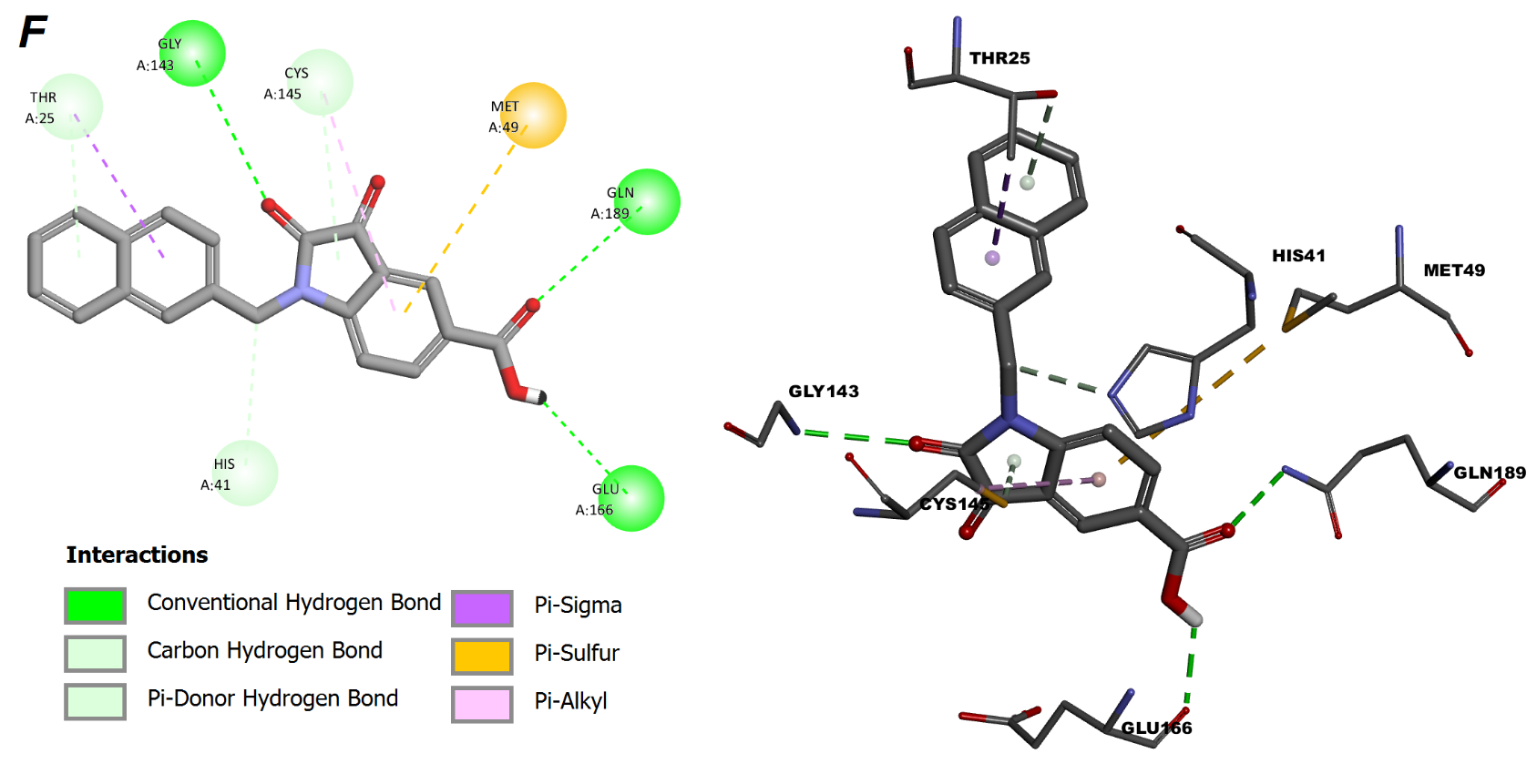


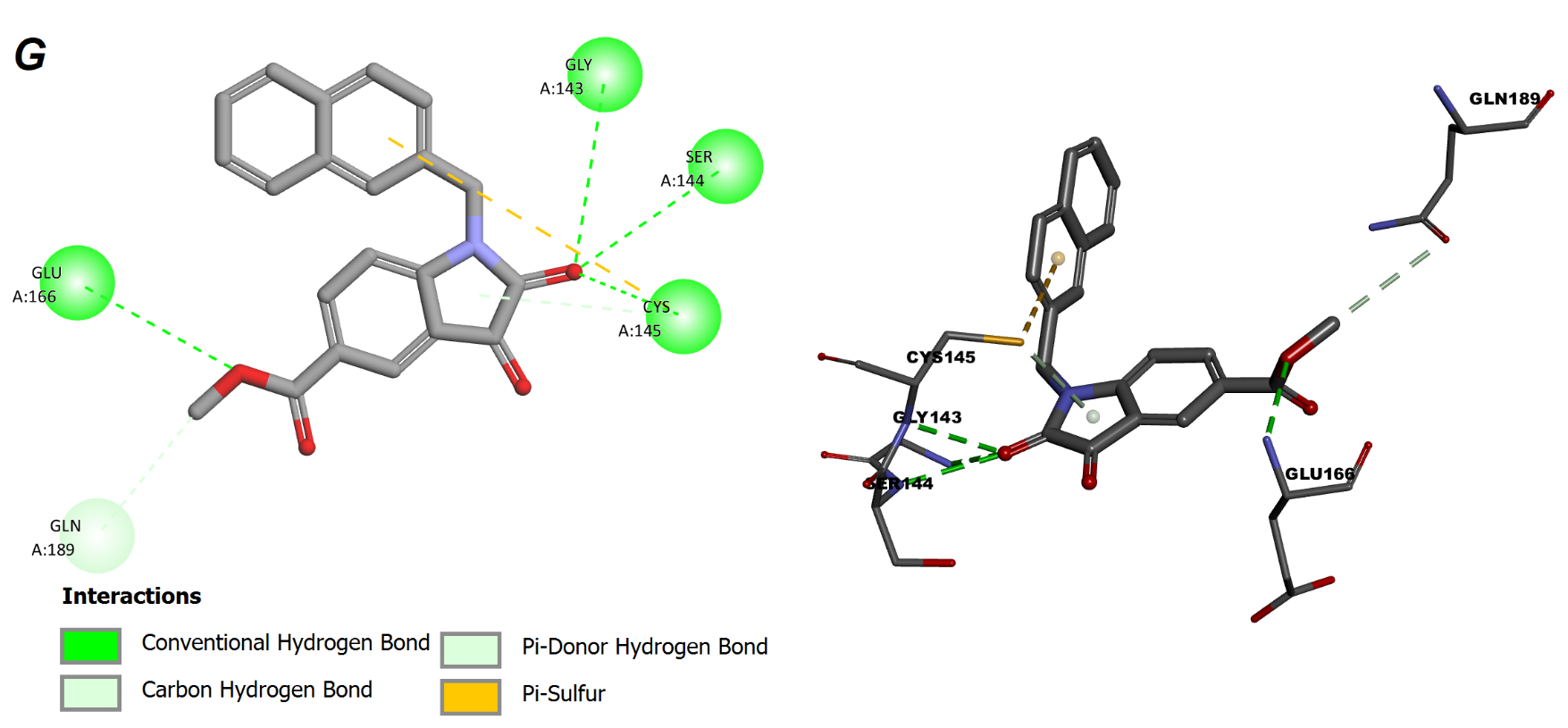


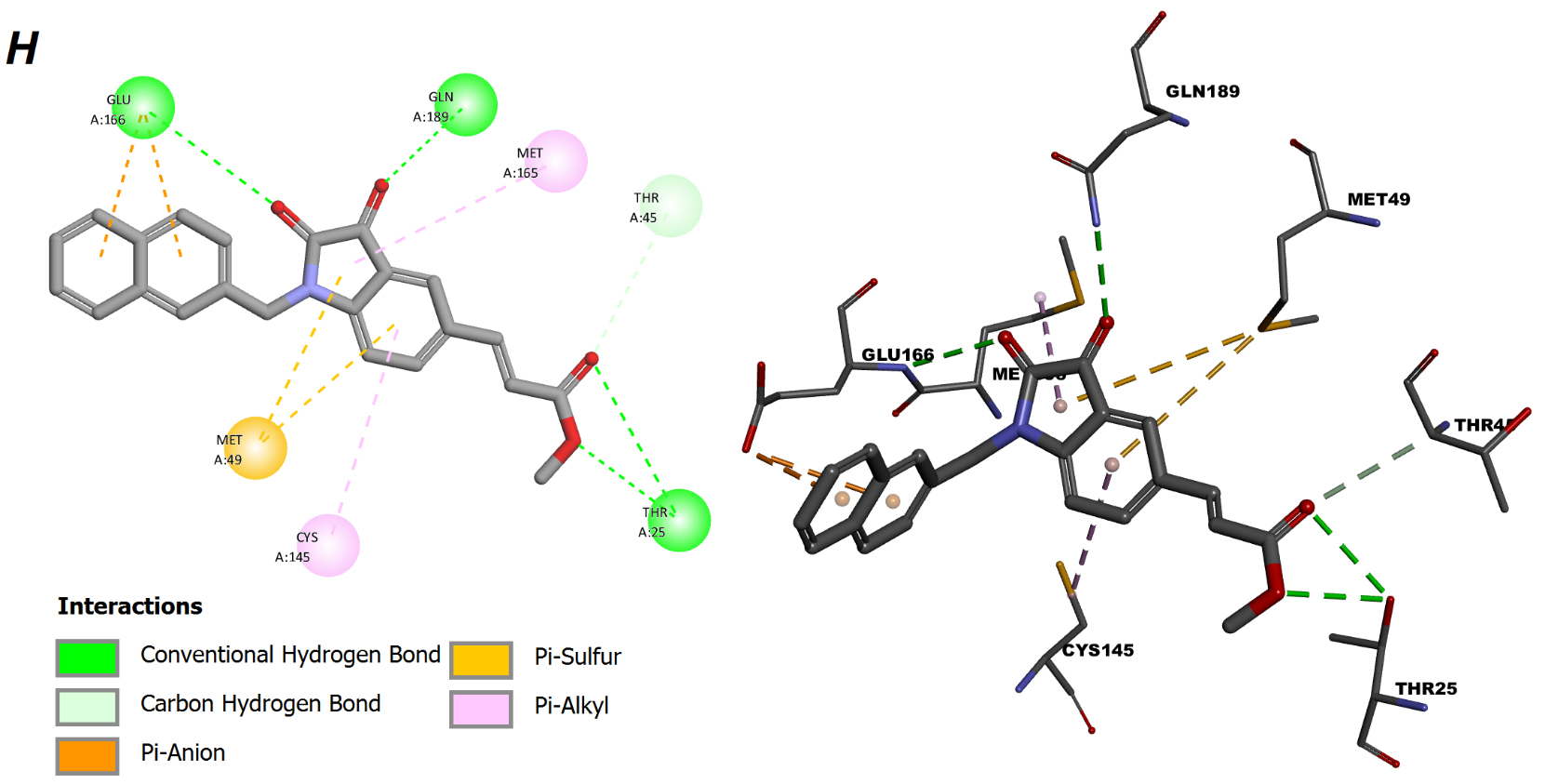


**Figure 2S.** Two and three‐dimensional diagram of (A) CHEMBL4524939 (B) CHEMBL4458417 (C) CHEMBL4452760 (D) CHEMBL4565907 (E) CHEMBL4443007 interactions with binding site residues of SARS-COV-1 3CLpro (6XHO) and (F) CHEMBL383761 (G) CHEMBL210543 (H) CHEMBL3103276interactions with binding site residues of SARS-COV-1 (1UK4)
